# Epigenetic Regulation of Hepatic Lipid Metabolism by DNA Methylation

**DOI:** 10.1002/advs.202206068

**Published:** 2023-06-06

**Authors:** Shirong Wang, Lin Zha, Xin Cui, Yu‐Te Yeh, Ruochuan Liu, Jia Jing, Huidong Shi, Weiping Chen, John Hanover, Jun Yin, Liqing Yu, Bingzhong Xue, Hang Shi

**Affiliations:** ^1^ Department of Biology Georgia State University Atlanta GA 30303 USA; ^2^ The Northern Medical District Chinese PLA General Hospital Beijing 100094 China; ^3^ Department of Internal Medicine University of Maryland School of Medicine Baltimore MD 21201 USA; ^4^ Department of Chemistry and the Center for Diagnosis and Therapeutics Georgia State University Atlanta GA 30303; ^5^ GRU Cancer Center and Department of Biochemistry and Molecular Biology Medical College of Georgia Augusta University Augusta GA 30912 USA; ^6^ Genomic Core Lab of National Institute of Diabetes and Digestive and Kidney Diseases National Institutes of Health Bethesda MD 20855 USA

**Keywords:** DNA methylation, epigenetics, hepatic steatosis

## Abstract

While extensive investigations have been devoted to the study of genetic pathways related to fatty liver diseases, much less is known about epigenetic mechanisms underlying these disorders. DNA methylation is an epigenetic link between environmental factors (e.g., diets) and complex diseases (e.g., non‐alcoholic fatty liver disease). Here, it is aimed to study the role of DNA methylation in the regulation of hepatic lipid metabolism. A dynamic change in the DNA methylome in the liver of high‐fat diet (HFD)‐fed mice is discovered, including a marked increase in DNA methylation at the promoter of Beta‐klotho (*Klb)*, a co‐receptor for the biological functions of fibroblast growth factor (FGF)15/19 and FGF21. DNA methyltransferases (DNMT) 1 and 3A mediate HFD‐induced methylation at the *Klb* promoter. Notably, HFD enhances DNMT1 protein stability via a ubiquitination‐mediated mechanism. Liver‐specific deletion of *Dnmt1* or *3a* increases *Klb* expression and ameliorates HFD‐induced hepatic steatosis. Single‐nucleus RNA sequencing analysis reveals pathways involved in fatty acid oxidation in *Dnmt1*‐deficient hepatocytes. Targeted demethylation at the *Klb* promoter increases *Klb* expression and fatty acid oxidation, resulting in decreased hepatic lipid accumulation. Up‐regulation of methyltransferases by HFD may induce hypermethylation of the *Klb* promoter and subsequent down‐regulation of *Klb* expression, resulting in the development of hepatic steatosis.

## Graphical abstract

High‐fat diet feeding enhances DNMTs’ mRNA and protein expression via increasing chromatin accessibility and decreasing ubiquitination‐mediated protein degradation, resulting in hypermethylation at the *Klb* promoter and subsequent downregulation of *Klb* expression; this in turn impairs fatty acid oxidation and oxidative phosphorylation and promotes the development of hepatic steatosis in mice.

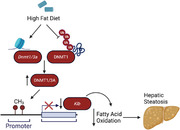

## 1 Introduction

Nonalcoholic fatty liver disease (NAFLD) is a growing metabolic disorder that has reached a high prevalence in US, with ≈10%–25% of adults being diagnosed in the general population.^[^ ^1^ ^]^ The prevalence of NAFLD increases up to 75% in patients with obesity, and therefore the disease has been proposed to be renamed as metabolic‐associated fatty liver disease.^[^ ^2^ ^]^ The hallmark of NAFLD is the excessive deposition of triglycerides (TG) in the liver.^[^ ^1^ ^]^ The disease begins with hepatic steatosis, a mild pathological change, and may progress to severe abnormalities such as nonalcoholic steatohepatitis (NASH), cirrhosis, and cancer.^[^ ^1^ ^]^

While extensive investigations have been devoted to the study of genetic pathways related to fatty liver diseases such as hepatic steatosis, fibrosis, and NASH, much less is known about epigenetic mechanisms underlying these disorders. Epigenetic regulation, including DNA methylation, links common environmental factors (e.g., diets) to complex diseases (e.g., metabolic disorders).^[^ ^3^ ^]^ DNA methylation of cytosines primarily at the CpG dinucleotides is one of the most common epigenetic modifications. CpG methylation frequently occurs in the promoters and 5’ ends of genes, thereby critically regulating gene transcription.^[^ ^4^ ^]^ De novo methylation of DNA is mainly handled by DNMT3A and 3B, while maintaining methylation patterns relies on another enzyme DNMT1 that catalyzes methylation on hemimethylated DNA strands during mitosis.^[^ ^4^ ^]^ However, substantial evidence also supports the role for DNMT1 in de novo methylation in non‐dividing cells.^[^ ^5^ ^]^ Meanwhile, DNA demethylation can be achieved by the ten‐eleven translocation (TET) dioxygenases that catalyze the hydroxylation of 5‐methylcytosine to form 5‐hydroxymethylcytosine and subsequent generation of 5‐formylcytosine and 5‐carboxylcytosine, which are then converted into unmodified cysteines by replication‐related dilution or glycosylation‐mediated base‐excision repair.^[^ ^6^ ^]^ DNA hyper‐methylation on the gene promoters is often associated with gene silencing, whereas DNA hypo‐methylation typically represents a transcriptionally active state.^[^ ^4^ ^]^

Metabolic disorders including obesity and NAFLD are complex diseases resulting from the interplay between genes and environmental factors, in which epigenetic mechanism serves as a link between the two.^[^ ^3^ ^]^ Increasing lines of evidence indicate that epigenetic regulation plays a key role in the development of metabolic diseases including NAFLD.^[^ ^7^, ^8^ ^]^ This is an evolving research area and yet much remains to be discovered on how DNA methylation regulates hepatic lipid metabolism and the development of fatty liver diseases. In the present study, we employed a comprehensive approach integrating a genome‐wide profiling of DNA methylation and gene expression, genetic models, and single‐nucleus RNA sequencing (snRNA‐Seq) analysis to delineate the role of DNA methylation in the regulation of hepatic lipid metabolism and to identify the key gene(s) whose DNA methylation status is epigenetically altered by the HFD, thereby contributing to the development of hepatic steatosis in male C57BL/6J mice.

## 2 Results

### 2.1 A Dynamic Change of the DNA Methylome in the Liver of HFD‐Fed Mice

**Figure 1:**
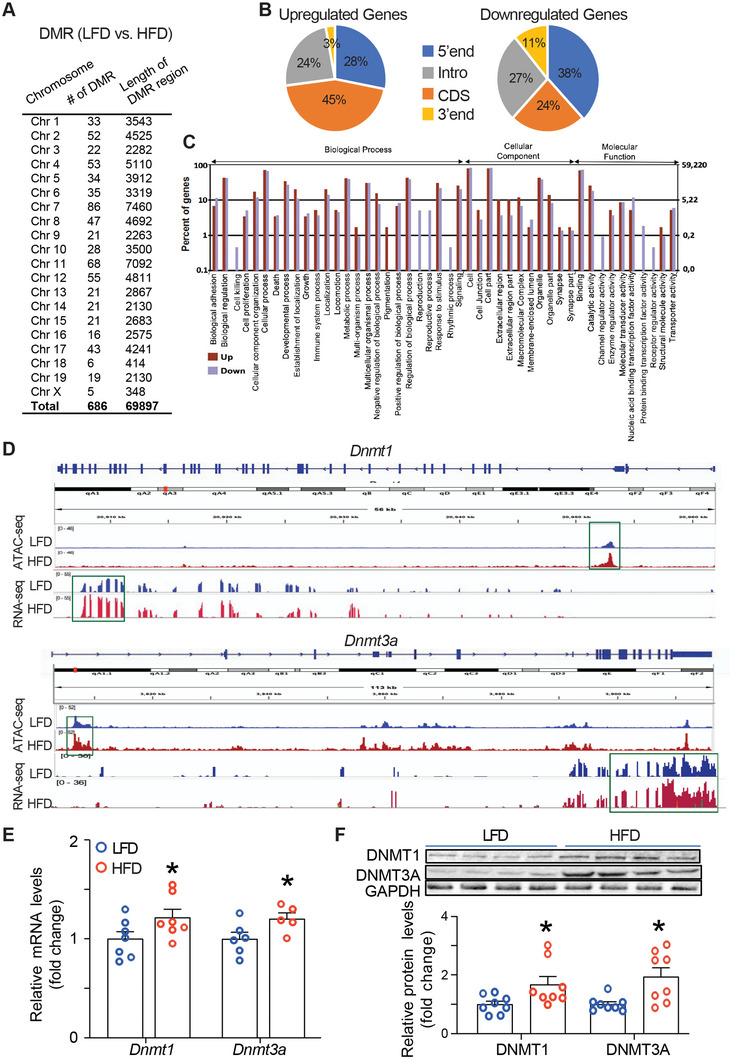
Dynamic changes of the DNA methylome in the liver of HFD‐fed mice. A) Differentially methylated regions (DMRs) in the liver of HFD‐ versus LFD‐fed mice. B) Methylation changes with either upregulation (left panel) or downregulation (right panel) occur in the gene body spanning from 5’‐end, coding sequence (CDS), intron, to 3’‐end. C) Genes with changes of DNA methylation are involved in various pathways. D) The association of the peaks of the chromatin accessibility at the *Dnmt1* promoter was analyzed by ATAC‐seq and the reads of the *Dnmt1* mRNA expression were analyzed by RNA‐seq (top panel); the association of the peaks of the chromatin accessibility at the *Dnmt3a* promoter analyzed by ATAC‐seq and the reads of the *Dnmt3a* mRNA expression analyzed by RNA‐seq (bottom panel). E) Quantitative RT‐PCR analysis of *Dnmt1* and *Dnmt3a* mRNA (n = 5‐7). (F) Immunoblotting analysis of DNMT1 and DNMT3A protein (*n* = 8). 6‐week‐old male C57BL/6J mice were fed either HFD or LFD for 12 weeks. All data are expressed as mean±SEM. *p < 0.05 versus LFD.

To profile the DNA methylome during the development of hepatic steatosis, we performed a genome‐wide DNA methylation analysis in the liver of C57BL/6J mice fed with either an HFD or low‐fat diet (LFD) using the Reduced Representation Bisulfite Sequencing (RRBS) approach. We first confirmed the establishment of the mouse model of hepatic steatosis by characterizing lipid accumulation in the liver. Biochemical analysis showed a marked increase in liver TG contents in HFD‐fed mice (Figure S1A, Supporting Information). Histological analysis by oil red O staining further confirmed lipid accumulation in hepatocytes, a feature of hepatic steatosis (Figure S1B, Supporting Information). The RRBS analysis revealed that there are up to 686 differentially methylated regions (DMRs) in HFD‐ versus LFD‐ fed mice (**Figure** **1**A). These DMRs cover 380 genes, including 298 genes whose methylation rates are up‐regulated by HFD (Table S1, Supporting Information, pages 1–7) and 82 genes whose methylation rates are down‐regulated by HFD (Table S1, Supporting Information, pages 8–9). The data suggest that HFD feeding mainly increases the DNA methylation on genes, which accounts for 78% of all genes with altered methylation rates (298/380). The methylation changes occur in the gene body spanning from 5’‐end, coding sequence (CDS), intron, to 3’‐end (Figure 1B). It is noteworthy that there are 119 genes with the methylation changes on the 5’‐end (Figure 1B), a known gene region whose methylation may influence gene transcription. Genes with methylation changes are involved in various pathways including biological process, cellular component, and molecular function (Figure 1C).

To determine which DNA methylation modifying enzymes mediate the HFD‐induced alterations on DNA methylation in the liver, we performed a comprehensive analysis of Assay for Transposase‐Accessible Chromatin Using Sequencing (ATAC‐seq) and RNA‐seq, which surveys genome‐wide chromatin accessibility and gene expression respectively, in the hopes that a gene with a concerted change of open chromatin accessibility and increased expression can be converged. Using the liver samples from HFD‐ and LFD‐fed mice, we compared the genome‐wide changes in chromatin landscape assessed by ATAC‐seq with the corresponding gene expression assessed by RNA‐seq and discovered a strong correlation between the chromatin accessibility status and the gene expression in *Dnmt1* and *Dnmt3a*. As shown in Figure 1D, ATAC‐seq analysis revealed an enhanced peak at the *Dnmt1* promoter (shown in green‐highlighted box) in the liver of HFD‐fed mice relative to LFD‐fed mice, indicative of increased chromatin accessibility at the *Dnmt1* promoter. This was associated with an up‐regulation of *Dnmt1* mRNA reads in the liver of HFD‐fed mice revealed by the RNA‐seq data (Figure 1D, lower panel). A similar trend of an open chromatin structure at the *Dnmt3a* promoter with increased gene expression reads was observed in HFD‐fed animals (Figure 1D, bottom panel). In support of this observation, we confirmed the enhanced expression of *Dnmt1* and *Dnmt3a* at both mRNA (Figure 1E) and protein (Figure 1F) levels in HFD‐fed mice by quantitative RT PCR and immunoblotting respectively. However, there were no changes of *Dnmt3b* mRNA and protein levels in the liver of HFD‐fed mice (Figure S2A,B, Supporting Information), nor were there any changes of chromatin accessibility and RNA‐seq reads at the *Dnmt3b* gene (Figure S2C, Supporting Information). These data suggest that DNA methylation may be important for the development of hepatic steatosis, and that DNMT1 and DNMT3A may be the key enzymes in this process.

### 2.2 DNA Methylation Regulates Hepatic Lipid Accumulation

To further determine the role of DNA methylation in the regulation of hepatic lipid metabolism, we employed a genetic approach by generating the mice with liver‐specific deletion of *Dnmt1* or *Dnmt3a* (LD1KO or LD3aKO) by an intravenous injection of AAV8‐TBG‐Cre virus, which has been successfully used in hepatocyte‐specific deletion of genes of interest.^[^ ^9^, ^10^ ^]^ We found that mRNAs and protein levels of *Dnmt1* and *Dnmt3a* decreased by more than 50% in the liver of LD1KO and LD3aKO mice respectively (Figure S3A,B, Supporting Information). To confirm the knockout efficiency of *Dnmt1* and *Dnmt3a* in hepatocytes, we isolated primary hepatocytes from LD1KO and LD3aKO mice and measured respective *Dnmt1* and *Dnmt3a* at mRNA and protein levels. We found that hepatocytes isolated from LD1KO mice had a reduction of *Dnmt1* mRNA by 60% and DNMT1 protein by 78% (Figure S4A, Supporting Information). A similar decrease of *Dnmt3a* mRNA and protein levels was also observed in primary hepatocytes isolated from LD3aKO mice (Figure S4B, Supporting Information).

**Figure 2:**
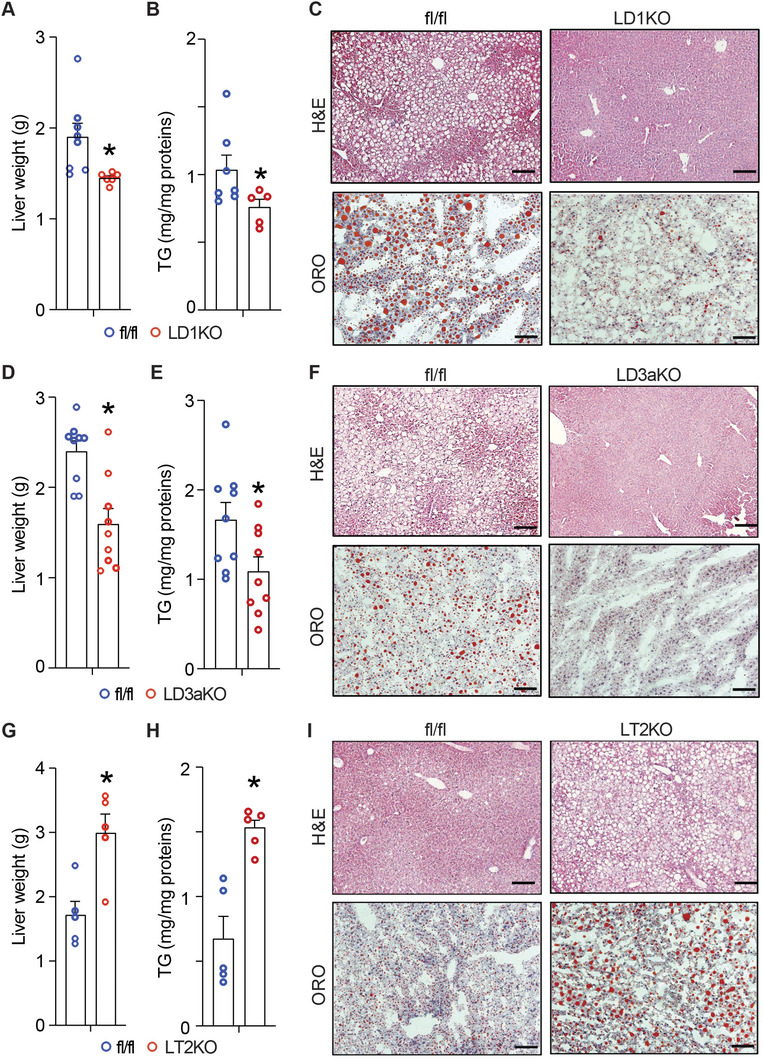
DNA methylation regulates hepatic lipid accumulation. A) Liver weight of LD1KO and fl/fl mice. B) Liver TG contents (normalized by protein contents) of LD1KO and fl/fl mice. (C) H&E or oil red O staining of the liver of LD1KO and fl/fl mice. D) Liver weight of LD3aKO and fl/fl mice. E) Liver TG contents (normalized by protein contents) of LD3aKO and fl/fl mice. F) H&E or oil red O staining of the liver of LD3aKO and fl/fl mice. G) Liver weight of LT2KO and fl/fl mice. H) Liver TG contents (normalized by protein contents) of LT2KO and fl/fl mice. I) Representative histology of the liver of LT2KO and fl/fl mice. *Dnmt1* fl/fl, *Dnmt3a* fl/fl, or *Tet2* fl/fl mice were intravenously injected with AAV‐TBG‐Cre virus to generate LD1KO, LD3aKO or LT2KO mice respectively, which were then fed with HFD for 10 weeks, 12 weeks and 8 weeks respectively. All data are expressed as mean±SEM. *n* = 5‐9; *p < 0.05 versus fl/fl.

We then put LD1KO mice on HFD for 10 weeks and conducted metabolic characterization. LD1KO mice had a slightly decreased body weight (Figure S5A, Supporting Information) without change in fat pad mass (Figure S5B, Supporting Information). Interestingly, LD1KO mice had significantly lower liver weight compared to the control flox/flox (fl/fl) mice receiving the AAV8‐TBG‐GFP virus (**Figure** **2**A), which was consistent with a reduced liver TG contents (Figure 2B). Histological analysis by H&E and oil red O staining further confirmed less hepatic lipid accumulation in LD1KO mice (Figure 2C). However, there was no difference in the circulating lipid profile including TG, total cholesterol (TC), and free cholesterol (FC) (Figure S5C, Supporting Information) between the two genotypes. We next conducted a metabolic characterization on LD3aKO mice fed HFD for 12 weeks. Similarly, there was a slight decrease in body weight (Figure S6A, Supporting Information) of LD3aKO mice without change in fat pad mass (Figure S6B, Supporting Information). Moreover, *Dnmt3a* deficiency in the liver reduced liver weight (Figure 2D) and TG contents (Figure 2E). Histological examination revealed a decrease in hepatic steatosis in LD3aKO mice (Figure 2F). Despite no change in circulating TG (Figure S6C, Supporting Information), LD3aKO mice exhibited reduced TC and FC contents in circulation (Figure S6C, Supporting Information). In sum, the data indicate that inhibiting DNA methyltransferases by the genetic approach ameliorates HFD‐induced hepatic steatosis in mice.

DNA demethylation can be made by a family of enzymes called the TETs including members TET1, TET2, and TET3 that are capable of removing the methyl group from 5‐methylcytosine.^[^ ^6^ ^]^ To study the physiological significance of TETs in the development of HFD‐induced hepatic steatosis, we first determined whether HFD feeding alters Tets’ mRNA expression in the liver of mice. Interestingly, 4‐week HFD feeding consistently inhibited the expression of *Tet1*, *Tet2*, and *Tet3* respectively (Figure S7, Supporting Information).

Using the liver samples from HFD‐ and LFD‐fed mice, we compared the genome‐wide changes in chromatin landscape assessed by ATAC‐seq with the corresponding gene expression assessed by RNA‐seq. While *Tet1* mRNA was barely detectable by the RNA‐seq analysis (Figure S8A, Supporting Information), we discovered a tendency of decreased expression of *Te2* and *Tet3* mRNA in the liver of HFD‐fed mice relative to LFD‐fed mice (Figure S8B,C, Supporting Information). Unlike the ATAC‐seq analysis that revealed increased chromatin accessibility at the promoters of *Dnmt1* and *Dnmt3a*, there was no significant change of chromatin landscapes at the genes of *Tets*, suggesting that mechanisms other than chromatin structural changes might be responsible for the down‐regulation of *Tets’* expression.

To further study the role of TETs in the development of hepatic steatosis, we knocked down all three *Tets* in the liver of mice. Intravenous injection of AAV Tet1‐3 shRNA achieved a reduction of *Tet1* mRNA by 75%, *Tet2* mRNA by 50%, and *Tet3* mRNA by 50% respectively without interfering nontargeted Tet expression (Figure S9A, Supporting Information). The animals were then challenged with HFD for 5 weeks. Among the three Tets, knocking down Tet2 exhibited the most significant effect on the liver phenotype evident by the most increased liver weight (Figure S9B, Supporting Information). This was consistent with a dramatic increase in the hepatic TG contents (Figure S9C, Supporting Information) and steatosis (Figure S9D, Supporting Information) in *Tet2* knockdown liver. To confirm the importance of TET2 in hepatic lipid metabolism, we further generated the mice with liver‐specific deletion of *Tet2* by intravenously injecting AAV8‐TBG‐Cre virus into *Tet2* fl/fl mice (LT2KO). Challenged with HFD for 8 weeks, LT2KO mice had a slightly increased body weight (Figure S10A, Supporting Information) and subcutaneous (SQ) fat pad mass (Figure S10B, Supporting Information). Further characterization of LT2KO mice discovered increased liver weight (Figure 2G) and TG contents (Figure 2H), which was consistent with the histological examination showing more lipid accumulation in LT2KO mice (Figure 2I). The hepatic steatosis observed in LT2KO mice was associated with increased circulating TC (Figure S10C, Supporting Information) and a trend of increase in FC and TG (Figure S10C, Supporting Information). In sum, these data indicate that LT2KO mice largely exhibit an opposite phenotype to those of LD1KO and LD3aKO mice, further underscoring the importance of DNA methylation in hepatic lipid metabolism.

### 2.3 DNA Methylation Regulates Fatty Acid Oxidation

**Figure 3:**
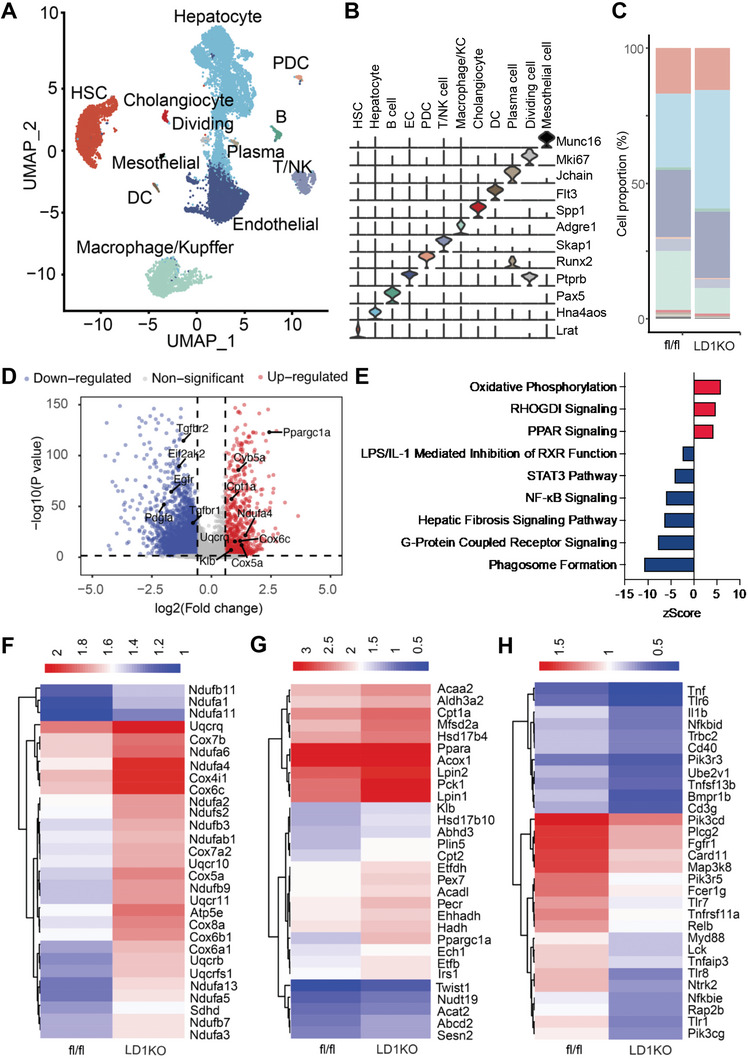
snRNA‐seq analysis reveals an up‐regulation of oxidative metabolism in Dnmt1‐deficient hepatocytes. (A) UMAP visualization shows the cell clusters including hepatocyte, endothelial cell (EC), hepatic stellate cell (HSC), macrophage/Kupffer cell (KC), plasmacytoid dendritic cell (PDC), dendritic cell (DC), T/NK cell, B cell (B), cholangiocyte, plasma cell (Plasma), mesothelial cell (Mesothelial), and dividing cell (Dividing), in the liver of LD1KO and fl/fl mice. (B) Violin plot of cell marker genes for each cell population. (C) Percentage of each cell population in total liver cells of LD1KO mice and fl/fl mice. (D) Volcano plot of differentially expressed genes in the hepatocytes of LD1KO mice versus fl/fl mice (fold change ≥1.5). (E) KEGG pathways that are up‐regulated or down‐regulated in the hepatocytes of LD1KO mice versus fl/fl mice. (F) Heatmap of gene expression of oxidative phosphorylation in the hepatocytes of LD1KO mice and fl/fl mice. (G) Heatmap of gene expression of fatty acid oxidation in the hepatocytes of LD1KO mice and fl/fl mice. (H) Heatmap of expression of the genes involved in the NF‐*κ*B pathway in the hepatocytes of LD1KO mice and fl/fl mice.

Liver is a heterogenous tissue that comprises of hepatocytes and non‐parenchymal cells. To delineate the cell type‐specific pathways underlying DNA methylation's effect on hepatic lipid metabolism, we performed a snRNA‐seq analysis for the frozen liver tissues from LD1KO mice and their fl/fl controls using the 10X genomics platform. After sequencing a total of 19 063 nuclei (6645 fl/fl and 12 451 LD1KO) and conducting an unbiased clustering with the Seurat R package, we identified 12 main cell populations in the liver including hepatocyte, endothelial cell (EC), hepatic stellate cell, macrophage/Kupffer cell (KC), plasmacytoid dendritic cell (PDC), dendritic cell (DC), T/NK cell, B cell, cholangiocyte, plasma cell, mesothelial cell and dividing cell (Figure **3**A), based on the known cell type markers^[^ ^11^ ^]^ (Figure 3B). We observed a shift in the relative composition of liver cell populations between LD1KO mice and fl/fl controls. Macrophages/Kupffer cells decreased from 25% in fl/fl liver to 9.4% in LD1KO mice, which was associated with a reduction of other immune cells, including T cells (from 5.3% to 3.2%) and DCs (from 2% to 1%), and ECs (from 37.5% to 28.8%) (Figure 3C). By contrast, hepatocytes accounted for more proportion of LD1KO liver cells, increasing up to 39% from 22% in fl/fl mouse liver cells (Figure 3C). These data suggest that *Dnmt1* deletion prevents HFD‐induced remodeling of the liver cell compositions by diminishing immune cell infiltration into the liver, thereby maintaining a healthy liver. Since the *Dnmt1* knockout was largely restricted to hepatocytes, we performed a bioinformatic analysis of gene expression patterns in hepatocytes. A volcano plot analysis disclosed 5552 genes that were differentially regulated (fold change ≥1.5), among which 1055 genes, including a panel of genes involved in oxidative phosphorylation such as *Ndufa1*, *Cox5a*, and *Cox6c*, and fatty acid oxidation such as *Pgc1α* and *Cpt1a*, were up‐regulated, and 4497 genes, including genes involved in fibrosis (e.g., *Tgfbr1*) and inflammation (e.g., *Eif2ak2*), were down‐regulated in Dnmt1‐deficient hepatocytes (Figure 3D). Further KEGG pathway analysis of the differentially expressed genes revealed 10 significantly up‐regulated pathways, among which oxidative phosphorylation was top‐ranked, and a dozen down‐regulated pathways, including the inflammatory pathway NF‐*κ*B and the hepatic fibrosis signaling pathway (Figure 3E), two hallmarks of NASH development. The results of the pathway analysis were in line with a hierarchical cluster analysis as shown in heatmaps indicating a marked up‐regulation of genes responsible for oxidative phosphorylation (Figure 3F) and fatty acid oxidation (Figure 3G) and a broad down‐regulation of genes involved in the NF‐*κ*B pathway (Figure 3H), phagosome formation pathway (Figure S11A, Supporting Information) and hepatic fibrosis signaling pathway (Figure S11B, Supporting Information) in *Dnmt1*‐knockout hepatocytes.

**Figure 4:**
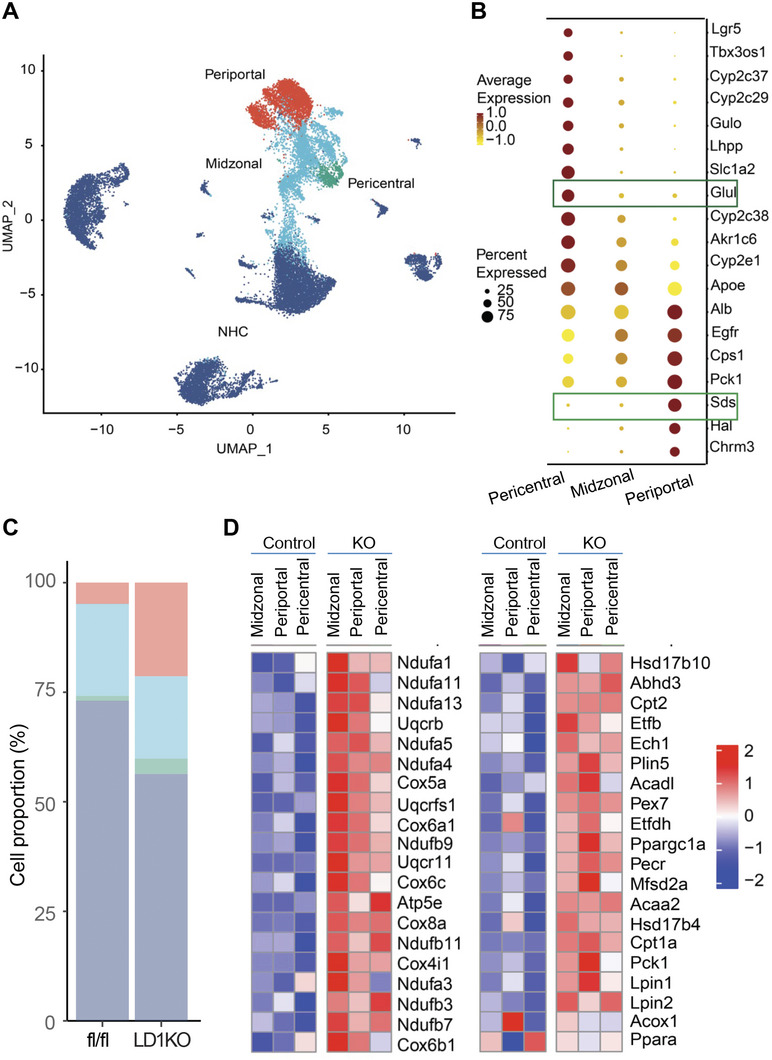
Dnmt1 deficiency promotes oxidative metabolism across hepatocyte zonation. A) UMAP visualization of hepatocyte zonation representing the periportal zone, midzonal zone (midzone), and pericentral zone. Nonhepatocytes: NHCs. B) Dot plot of differentially expressed zonation marker genes across the three zones. C) The proportion of each hepatocyte subpopulation in the total liver cells of LD1KO mice and fl/fl mice. D) Heatmap of gene expression of oxidative phosphorylation across the three hepatocyte zones in LD1KO mice and fl/fl mice (left panel) and heatmap of gene expression of fatty acid oxidation across the three hepatocyte zones in LD1KO mice and fl/fl mice (right panel).

Hepatocytes that align along the lobule axis display a zonation pattern with a spatial heterogeneity in both gene expression and metabolic functions.^[^ ^12^, ^13^ ^]^ We therefore sought to examine the hepatic gene expression in a spatial resolution. We identified 3 sub‐clusters of hepatocytes (**Figure** **4**A) based on the expression of hepatocyte zonation markers (Figure 4B)^[^ ^14^ ^]^ and named them as periportal zone, midzonal zone (midzone), and pericentral zone as previously defined.^[^ ^12^ ^]^ Unlike the zonation marker genes Sds and Glul that display a distinct expression in the periportal and pericentral zone respectively, no such specific landmark genes can be found in our study for the midzone presumably due to its feature as a transition region with gradients of hepatocyte gene expression across all three zones (Figure 4B).^[^ ^15^ ^]^ Interestingly, the periportal hepatocytes, featured by their superb abilities to utilize fatty acids due to possession of oxidatively active mitochondria,^[^ ^15^ ^]^ increased dramatically in their proportion accounting for the total LD1KO liver cells, compared to that of fl/fl mice (Figure 4C). Further hierarchical cluster analysis showed a marked up‐regulation of genes responsible for oxidative phosphorylation (Figure 4D left panel) and fatty acid oxidation (Figure 4D right panel) across the three zones in the LD1KO hepatocytes. Interestingly, the midzonal hepatocytes in LD1KO mice appeared to have a conspicuous increase in the expression of oxidative phosphorylation genes (Figure 4D left panel), suggesting a functional transition to the periportal hepatocytes featured by a strong capacity for oxidative metabolism. In sum, these data indicate that enhanced fatty acid oxidation may be responsible for the reduced hepatic steatosis in LD1KO mice.

**Figure 5:**
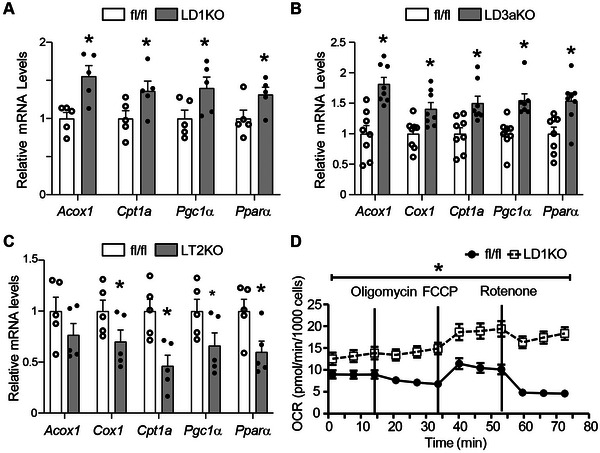
DNA methylation regulates the expression of fatty acid oxidative genes. A) Quantitative RT‐PCR analysis of fatty acid oxidative gene expression in the liver of LD1KO and fl/fl mice. B) Quantitative RT‐PCR analysis of fatty acid oxidative gene expression in the liver of LD3aKO and fl/fl mice. C) Quantitative analysis of fatty acid oxidative gene expression in the liver of LT2KO and fl/fl mice. D) Oxygen consumption rate (OCR) of primary hepatocytes isolated from LD1KO and fl/fl mice. *Dnmt1* fl/fl, *Dnmt3a* fl/fl, or *Tet2* fl/fl mice were intravenously injected with AAV‐TBG‐Cre virus to generate LD1KO, LD3aKO or LT2KO mice respectively, which were then fed with HFD as described in Figure 2. All data are expressed as mean±SEM. *n* = 5‐8; *p < 0.05 versus fl/fl.

Indeed, quantitative PCR analysis further confirmed that inhibiting DNMT1 in hepatocytes significantly promoted the expression of genes involved in fatty acid oxidation such as carnitine palmitoyl transferase 1(*Cpt1)*, acyl‐coa oxidase 1 (*Acox1)*, peroxisome proliferator‐activated rexeptor‐gamma coactivator 1 alpha (*Pgc1α)*, and peroxisome proliferator‐activated receptor alpha (*Pparα)* (**Figure** **5**A), without change of lipogenic gene expression Figure S12A, Supporting Information). Similarly, LD3aKO mice exhibited increased expression of fatty acid oxidative genes (Figure 5B) without change of most lipogenic gene expression except a decreased expression of acetyl‐coa carboxylase 1 (*Acc1)* and fatty acid synthase (Figure S12B, Supporting Information). In contrast, LT2KO mice with *Tet2* deletion in hepatocytes had a down‐regulation of fatty acid oxidative gene expression (Figure 5C) with a reciprocal up‐regulation of lipogenic gene expression such as *Acc1* and stearoyl‐coa desaturase 1 and fatty acid transporter gene cluster of differentiation 36 in the liver (Figure S12C, Supporting Information). In support of altered DNA methylation that regulates the fatty acid oxidative program, seahorse analysis revealed an upregulation of oxygen consumption rate (OCR) in *Dnmt1*‐deficient hepatocytes isolated from LD1KO mice (Figure 5D). In sum, these data indicate that regulation of fatty acid oxidation by DNA methylation might be important for the development of HFD‐induced hepatic steatosis.

### 2.4 Methylation at the Klb Promoter is Enhanced by HFD Feeding

**Figure 6:**
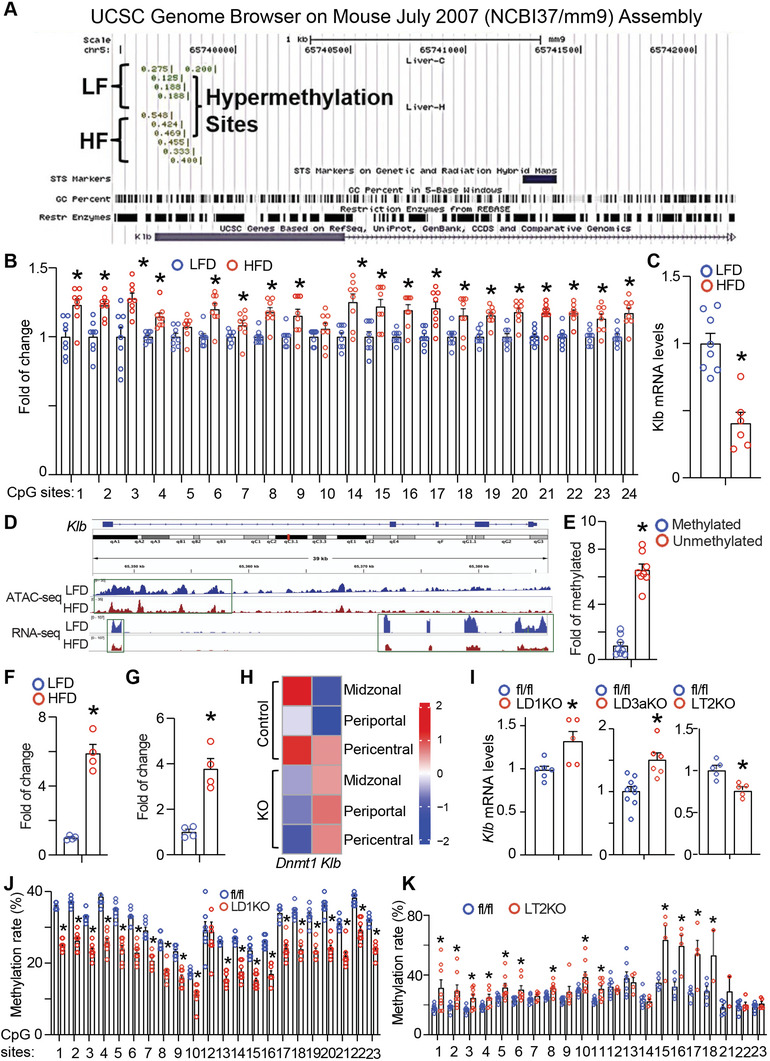
Methylation of the *Klb* promoter is enhanced by HFD. A) RRBS analysis reveals an enhanced DNA methylation rate at the *Klb* promoter by HFD as shown in UCSC Genome Browser on Mouse (NCBI37/mm9) Assembly. B) Pyrosequencing analysis of the DNA methylation at the CpG sites at the *Klb* promoter in the liver of HFD‐ and LFD‐fed mice (*n* = 8). C) Quantitative RT‐PCR analysis of *Klb* mRNA expression in liver of HFD‐ and LFD‐fed mice (*n* = 6–8). D) The association of the peaks of the chromatin accessibility at the *Klb* promoter was analyzed by ATAC‐seq and the reads of the *Klb* mRNA expression analyzed by RNA‐seq in the liver of HFD‐ and LFD‐fed mice. () Luciferase activity of the *Klb* promoter regulated by DNA methylation (*n* = 8). F) DNMT1 binding to the *Klb* promoter was measured by ChIP assays (*n* = 4). G) DNMT3A binding to the *Klb* promoter measured by ChIP assays (*n* = 4). 6‐week‐old male C57BL/6J mice were fed either LFD or HFD for 12 weeks. All data are expressed as mean±SEM. *p < 0.05 versus LFD. H) Heatmap of *Klb* and *Dnmt1* expression across the three hepatocyte zones (periportal zone, midzone, and pericentral zone) in LD1KO mice and fl/fl mice with snRNA‐seq analysis. I) Quantitative RT‐PCR analysis of *Klb* mRNA expression in *Dnmt1*‐deficient liver (left), *Dnmt3a*‐deficient liver (middle), or *Tet2*‐deficient liver (right) (*n* = 5‐9). J) Pyrosequencing analysis of DNA methylation rate in the CpG sites of the Klb promoter in the liver of LD1KO mice and fl/fl mice (*n* = 8). K) Pyrosequencing analysis of DNA methylation rate in the CpG sites of the Klb promoter in the liver of LT2KO mice and fl/fl mice (*n* = 4‐8). All data are expressed as mean ± SEM. *p < 0.05 versus fl/fl.

To narrow down the molecules whose down‐regulations are direct targets of promoter hypermethylation due to HFD feeding, we analyzed our RRBS data and discovered 83 genes with hypermethylation at 5’‐end, a known gene region whose altered methylation most likely influence gene transcription. We then prioritized the genes that have been known to be involved in signal transduction and transcriptional regulation in metabolism, which led to 16 genes (*Klb, Ctsb, Dusp26, Lgals3, Gdf10, Onecut1, Mgat1, Agap2, Aph1a, Lhcgr, Prom2, Mamstr, Crb2, Ub15, Actg, and Palm3*). We reasoned that if the genes are epigenetic targets for the development of hepatic steatosis, HFD feeding ought to alter their DNA methylation, resulting in changes of gene expression. We therefore measured the expression of the 16 genes with changes of DNA methylation at the 5’‐end, which are also involved in metabolism, in the hope that a gene with a concerted change of increased DNA methylation at the 5’‐end and decreased gene expression can be converged. Among the 16 genes we screened with quantitative RT PCR, the expression of some genes such as *Dusp26, Mgat1, Lhcgr, Prom2*, and *Ub15* was hardly detectable in the liver (data not shown), while there was no difference in the expression of *Ctsb, Gdf10, Onecut1*, and others between HFD‐fed and LFD‐fed mice (representative data of gene expression was shown in Figure S13, Supporting Information). *Klb* was left as the only hit that fits the pattern with increased DNA methylation at the 5’‐end and decreased gene expression. Specifically, we discovered that the 5’‐end methylation rate of the *Klb* promoter was significantly increased in the liver of HFD‐fed mice as indicated in the UCSC Genome Browser (**Figure** **6**A). KLB has been identified as a coreceptor necessary for the physiological functions of FGF15/19 and FGF21, two hormones involved in various metabolic pathways including hepatic lipid metabolism.^[^ ^16^, ^17^ ^]^ The proximal promoter and 5’ region of *Klb* is enriched with CpG islands (Figure S14, Supporting Information), suggesting a possibility that the *Klb* promoter is subject to the regulation of DNA methylation. We therefore further conducted pyrosequencing to determine the methylation status of the CpG sites, which are located within the downstream proximity of the TATA box at the *Klb* promoter and at the beginning of the first exon. Indeed, our pyrosequencing analysis confirmed that HFD feeding significantly increased DNA methylation at the CpG sites at the *Klb* promoter (Figure 6B). This was associated with downregulation of *Klb* mRNA expression in the liver of HFD‐fed mice analyzed by quantitative PCR (Figure 6C), suggesting that enhanced DNA methylation at the *Klb* promoter may inhibit its gene expression. Changes in DNA methylation have been shown to modulate histone modifications, which may act cooperatively to influence chromatin structure and thereby regulate gene expression.^[^ ^18^ ^]^ Indeed, ATAC‐seq analysis revealed a reduced chromatin accessibility at the *Klb* promoter in the liver of HFD‐fed mice, which was associated with a downregulation of *Klb* mRNA reads revealed by RNA‐seq data (Figure 6D). To determine whether the *Klb* promoter is indeed regulated by methylation, we cloned a 700 bp *Klb* proximal promoter including the CpG‐enriched region into pGL3‐luciferase expression vector. We then examined the fully methylated versus unmethylated *Klb* promoter activity. Our luciferase assays showed that the luciferase activity of the unmethylated promoter was fivefold higher than that of the fully methylated promoter (Figure 6E). We next determined whether the *Klb* promoter may serve as a target for DNMT1 or DNMT3A to regulate its methylation and expression. We conducted ChIP assays followed by SYBR green quantitative PCR to examine DNMTs’ binding to the *Klb* promoter. We found that HFD feeding significantly increased DNMT1 (Figure 6F) or DNMT3A (Figure 6G) binding to the *Klb* promoter in the liver of mice. Unbiased snRNA‐seq analysis of hepatocyte gene expression discovered an up‐regulation of the *Klb* mRNA in *Dnmt1*‐deficient hepatocytes across the three zones as shown in the heatmap with down‐regulated *Dnmt1* expression as a control (Figure 6H). Indeed, *Klb* mRNA was up‐regulated in *Dnmt1*‐ or *Dnmt3a*‐deficient liver while down‐regulated in *Tet2* knockout liver (Figure 6I). Deletion of *Dnmt1* reduced the average DNA methylation rates in almost all CpG sites at the *Klb* promoter (Figure 6J), whereas deletion of *Tet2* increased the average DNA methylation in most CpG sites at the *Klb* promoter (Figure 6K). Signal network analysis using the differentially expressed genes of the *Dnmt1*‐deficient hepatocytes from the snRNA‐seq dataset revealed KLB as a metabolic nexus that governs the signals integrating mitochondrial oxidative phosphorylation and hepatic fatty acid oxidation (Figure S15, Supporting Information). For instance, the FGF15/FGF21/KLB axis may regulate mitochondrial oxidative phosphorylation through the upregulation of PGC1*α*, a master regulator of mitochondrial biogenesis. Meanwhile, the FGF15/FGF21/KLB signal may also promote fatty acid oxidation by cooperating with the nuclear receptor PPAR*α* that activates the transcriptional program of the fatty acid oxidative genes.

**Figure 7:**
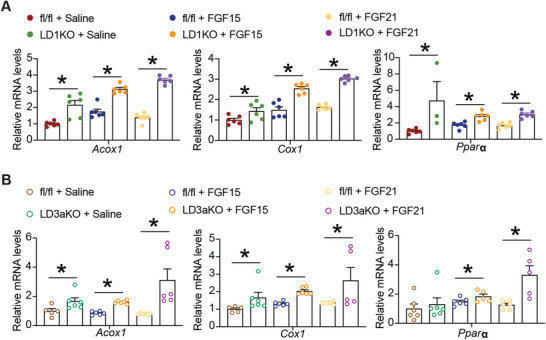
*Dnmt1* or *Dnmt3a* deficiency increases FGF15 or FGF21‐stimulated fatty acid oxidative gene expression in primary hepatocytes. A) Quantitative RT‐PCR analysis of fatty acid oxidative gene expression in the *Dnmt1* deficient hepatocytes (*n* = 3‐6). B) Quantitative RT‐PCR analysis of fatty acid oxidative gene expression in the *Dnmt3a* deficient hepatocytes (*n* = 5‐6). Primary hepatocytes were isolated from *Dnmt1*‐ or *Dnmt3a*‐floxed mice and were infected with AAV8‐TBG‐Cre AAV or control virus to generate *Dnmt1* or *Dnmt3a*‐deficient hepatocytes. The hepatocytes were then treated with FGF15 or FGF21 for 4 h. All data are expressed as mean±SEM. *p < 0.05 versus fl/fl.

To test whether FGF15 or FGF21 has a direct impact on lipid metabolism in hepatocytes with *Dnmt1* or *Dnmt3a* deficiency, we generated *Dnmt1* or *Dnmt3a* deficient hepatocytes by infecting primary hepatocytes from *Dnmts*‐floxed mice with AAV8‐TBG‐Cre virus and treated them with FGF15 or FGF21. Quantitative RT PCR analysis revealed that *Dnmt1* or *Dnmt3a* deficiency increased FGF15 or FGF21‐stimulated fatty acid oxidative gene expression (**Figure** **7**A,B). These data strongly suggest that DNMT1 and DNMT3A may act on the *Klb* promoter to promote DNA methylation in response to HFD, resulting in HFD‐induced hepatic steatosis.

### 2.5 Specific Demethylation at the *Klb* Promoter Ameliorates Hepatic Steatosis

Although we have determined the roles of DNMT1, DNMT3a, and TET2 in the regulation of *Klb* promoter methylation and lipid metabolism, it is not clear whether specific methylation at the *Klb* promoter mediates these metabolic changes in the liver of LD1KO, LD3aKO, and LT2KO mice. Besides, DNMT1 and 3A potentially catalyze DNA methylation in many genes other than Klb and deletion of the DNA methylation enzymes inevitably has an impact on the global DNA methylation status, which may potentially confound the metabolic phenotypes. We therefore adopted a modified clustered regularly interspaced short palindromic repeats (CRISPR)/RNA‐guided system to induce gene‐specific demethylation at the *Klb* promoter, guided by sequence‐specific single guide RNAs (sgRNAs).^[^ ^19^ ^]^

**Figure 8:**
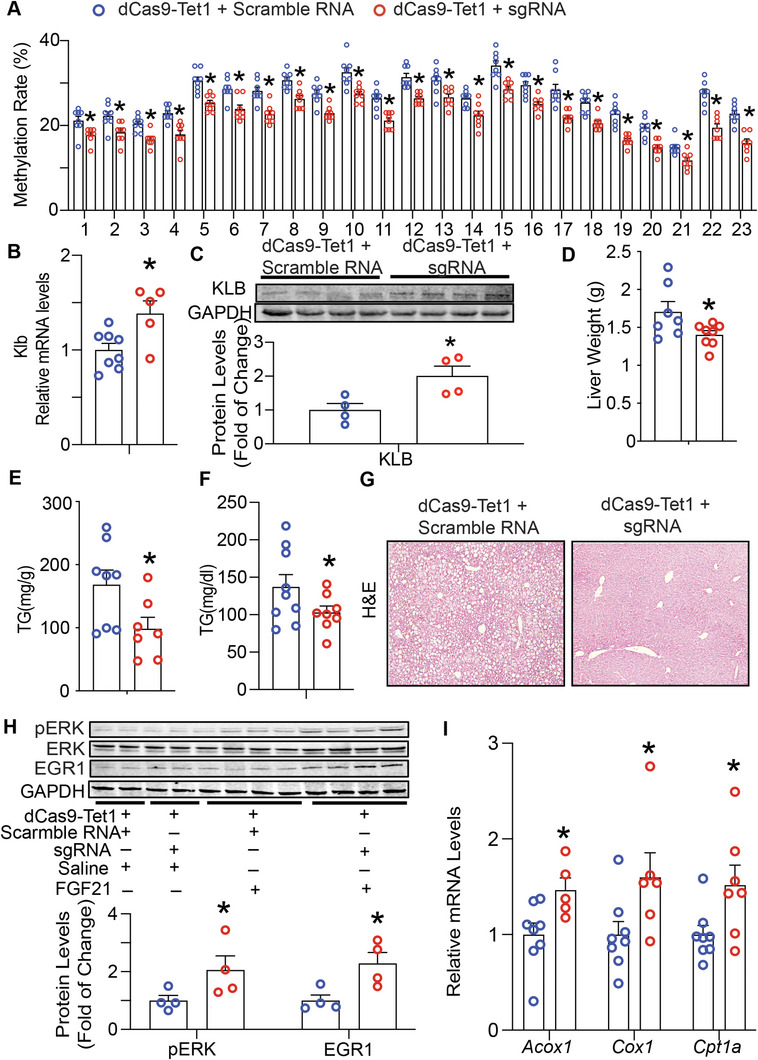
Reducing DNA methylation at the *Klb* promoter in liver ameliorates hepatic steatosis. A) Pyrosequencing analysis of DNA methylation rate in the CpG sites of the Klb promoter in the liver of mice infected with dCas9‐Tet1 and sgRNA lentivirus or dCas9‐Tet1 and scramble RNA lentivirus (*n* = 7‐8). B) Quantitative RT‐PCR analysis of *Klb* mRNA expression in the liver of mice infected with dCas9‐Tet1 and sgRNA lentivirus or dCas9‐Tet1 and scramble RNA lentivirus (*n* = 5‐8). C) Immunoblotting analysis of KLB protein in the liver of mice infected with dCas9‐Tet1 and sgRNA lentivirus or dCas9‐Tet1 and scramble RNA lentivirus (*n* = 4). D) Liver weight of mice infected with dCas9‐Tet1 and sgRNA lentivirus or dCas9‐Tet1 and scramble RNA lentivirus (*n* = 7‐8). E) Liver TG contents of LD1KO and fl/fl mice infected with dCas9‐Tet1 and sgRNA lentivirus or dCas9‐Tet1 and scramble RNA lentivirus (*n* = 7‐8). F) Circulating TG levels of mice infected with dCas9‐Tet1 and sgRNA lentivirus or dCas9‐Tet1 and scramble RNA lentivirus (*n* = 8). G) Representative histology of liver of mice infected with dCas9‐Tet1 and sgRNA lentivirus or dCas9‐Tet1 and scramble RNA lentivirus. H) Immunoblotting analysis of phosphorylation of ERK1 and EGR1 activated by FGF21 in the liver of mice infected with dCas9‐Tet1 and sgRNA lentivirus or dCas9‐Tet1 and scramble RNA lentivirus (*n* = 2‐4). I) Quantitative RT‐PCR analysis of fatty acid oxidative gene expression in liver of mice infected with dCas9‐Tet1 and sgRNA lentivirus or dCas9‐Tet1 and scramble RNA lentivirus (*n* = 5‐8). 7‐week old C57BL/6J male mice were intravenously injected with dCas9‐Tet1 and sgRNA lentivirus or dCas9‐Tet1 and scramble RNA lentivirus and the mice were then fed with HFD for 8 weeks. All data are expressed as mean±SEM. *p < 0.05 versus scramble RNA virus.

Male C57BL/6J mice were injected with lentiviral mixtures expressing deactivated CRISPR‐associated protein 9 (dCas9)‐Tet1 and sgRNA or scramble non‐targeting sgRNA as a control intravenously, and mice were put on HFD one week after lentiviral injection. Mice infected with dCas9‐Tet1 and sgRNA lentivirus did not show any difference in body weight and fat pad mass compared to the controls (Figure S16A,B, Supporting Information). Lentiviral dCas9‐Tet1 and sgRNA effectively reduced the average DNA methylation rate in almost all CpG sites at the *Klb* promoter (**Figure** **8**A), which was associated with an up‐regulation of *Klb* mRNA and protein expression (Figure 8B,C). Moreover, injection of dCas9‐Tet1/sgRNA lentivirus significantly reduced liver weight and TG content in mice fed HFD (Figure 8D,E). As a result, circulating TG levels were also decreased in mice infected with dCas9‐Tet1 lentivirus (Figure 8F). Further histological examination revealed reduced hepatic steatosis in the mice injected with dCas9‐Tet1/sgRNA lentivirus compared to those infected with the control virus (Figure 8G). Since Klb is a coreceptor required for a proper FGF21 signaling, the mice infected with dCas9‐Tet1/sgRNA or the control lentivirus were injected with FGF21 to examine its signaling. dCase9‐Tet1/sgRNA infected mice displayed an enhanced phosphorylation of extracellular signal‐regulated kinase 1 and early growth response 1 induced by FGF21 compared to the control mice (Figure 8H). This was consistent with an up‐regulation of fatty acid oxidative gene expression including *Cpt1α*, *Acox1*, and cytochrome c oxidase 1 (Figure 8I), although there was no difference in lipogenic gene expression (Figure S16C, Supporting Information). Since FGF15/19 has been shown to repress bile acid synthesis, we therefore measured the expression of genes involved in bile acid synthesis. Quantitative RT‐PCR analysis did not show a difference in the expression of genes including cytochrome P450 (*Cyp*)*7a1*, *Cyp8b1*, and *Cyp27a1* between dCas9‐Tet1 virus‐infected mice and their controls (Figure S16D, Supporting Information). In addition, dCas9‐Tet1 virus‐infected mice did not exhibit any change in the expression of FGF21 in the liver (Figure S16E, Supporting Information).

Using the same modified CRISPR/RNA‐guided system, we conducted a gain‐of‐function experiment to induce specific methylation at the *Klb* promoter with lentiviral mixtures expressing dCas9‐Dnmt3a and sgRNA. Male C57BL/6J mice were injected with lentiviral mixtures expressing dCas9‐Dnmt3a and sgRNA or scramble non‐targeting sgRNA as a control intravenously, and mice were put on HFD 1 week after lentiviral injection. Mice infected with dCas9‐Dnmt3a and sgRNA lentivirus had a slight increase in body weight (Figure S17A, Supporting Information). Lentiviral dCas9‐Dnmt3a and sgRNA effectively increased the DNA methylation rates at the *Klb* promoter (Figure S17B, Supporting Information), which was associated with a down‐regulation of *Klb* mRNA expression (Figure S17C, Supporting Information). Moreover, injection of dCas9‐Tet1/sgRNA lentivirus increased liver weight and TG content in mice fed HFD (Figure S17D,E, Supporting Information), which was associated with increased TG levels in circulation (Figure S17F, Supporting Information). Further histological examination revealed a marked increase in hepatic steatosis in the mice injected with dCas9‐Dnmt3a/sgRNA lentivirus compared to those infected with the control virus (Figure S17G, Supporting Information).

Taken together, our data indicate that inhibiting methylation directly at the *Klb* promoter promotes hepatic FGF15/FGF21/KLB signaling and subsequent fatty acid oxidation, ameliorating hepatic steatosis in mice.

### 2.6 Regulation of Hepatic DNMT1 Protein Stability by HFD

**Figure 9:**
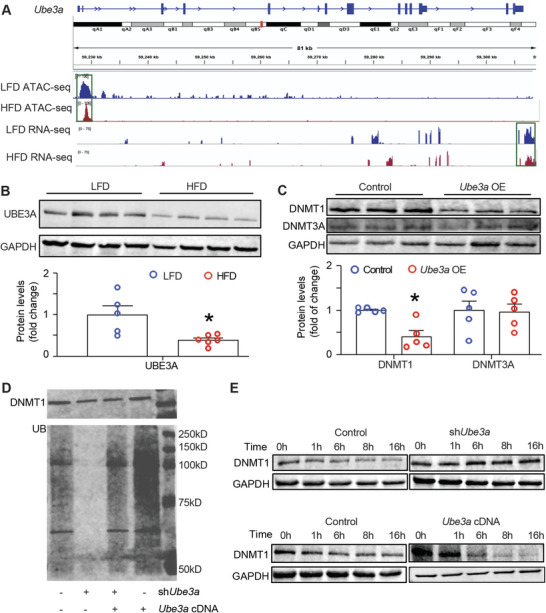
Regulation of DNMT1 protein stability by UBE3A. A) The association of the peaks of the chromatin accessibility at the *Ube3a* promoter analyzed by ATAC‐seq and the reads of the *Ube3a* mRNA expression analyzed by RNA‐seq in the liver of HFD‐fed or LFD‐fed mice. B) Immunoblotting analysis of UBE3A in the liver of mice fed LFD or HFD. All data are expressed as mean ± SEM. *n* = 5; *p < 0.05 versus LFD. C) Immunoblotting analysis of DNMT1 and DNMT3A protein in the liver of mice infected with AAV *Ube3a* expression virus. All data are expressed as mean ± SEM. *n* = 5; *p < 0.05 versus control. D) UBE3A regulation of DNMT1 ubiquitination. *Ube3a* was knocked down by shRNA or re‐expressed into the knockdown cells with *Ube3a* expression vectors or overexpressed with *Ube3a* expression vectors in HEK293 cells. DNMT1 protein was immunoprecipitated with an anti‐DNMT1 antibody and followed by immunoblotting with an anti‐ubiquitin antibody. E) UBE3A promotes DNMT1 protein degradation in a cycloheximide (CHX) chase assay. The CHX chase assay was conducted in the HEK293 cells with *Ube3a* knockdown or overexpression. The cells were pre‐treated with CHX and then harvested in a time course ranging from 0 to 16 h.

Since HFD appeared to exert a more potent effect on DNMT1 and DNMT3A protein levels than their respective mRNA levels, we reasoned that DNMT1 and DNMT3A protein might be regulated by protein stability. Recently the E3 ligase ubiquitin‐protein ligase E3A (UBE3A) has been reported to prevent HFD‐induced hepatic steatosis in mice.^[^ ^20^ ^]^ Interestingly, ATAC‐seq analysis revealed reduced peak reads at the Ube3a promoter in the liver of HFD‐fed mice, which was associated with decreased *Ube3a* mRNA reads revealed by RNA‐seq data (**Figure** **9**A). This was consistent with a down‐regulation of UBE3A protein levels in the liver of HFD‐fed mice (Figure 9B). We then assessed the role of UBE3A in the regulation of DNMT1 protein stability. We found that overexpressing UBE3A in the liver of the C57Bl/6J male mice infected with AAV8 virus carrying *Ube3a* expression constructs significantly reduced DNMT1 protein levels while without effect on DNMT3A protein levels (Figure 9C). We next examined DNMT1 ubiquitination with gain‐ or loss‐ of *Ube3a* in HEK293 cells. HEK293 cells with silenced or forced expression of *Ube3a* were treated with proteasome inhibitor MG132 to accumulate ubiquitinated proteins. We found that knocking down *Ube3a* in HEK293 cells abolished the ubiquitination of DNMT1 protein, which was largely restored by re‐introducing the Ube3a expression vector into the knockdown cells (Figure 9D). In contrast, overexpressing *Ube3a* significantly increased DNMT1 ubiquitination (Figure 9D). We further confirmed UBE3A regulation of DNMT1 protein stability in a cycloheximide (CHX) chase assay. Cells were pretreated with CHX, a ribosome inhibitor, to suppress DNMT1 protein synthesis. Inactivating *Ube3a* by shRNA knockdown maintained high levels of DNMT protein at the late time point of 16 hours while overexpression *Ube3a* markedly reduced DNMT1 protein at the early time point of 6 h (Figure 9E). These data suggest that DNMT1 protein stability was regulated by an UBE3A‐mediated ubiquitination and that HFD feeding may enhance DNMT1 protein levels in the liver via inhibiting Ube3a expression.

## 3 Discussion

NAFLD is a serious metabolic disorder that has reached a high prevalence in US.^[^ ^1^ ^]^ While numerous studies have been devoted to the investigation of genetic pathways related to the fatty liver disease, much is unknown about the role of epigenetic regulation in this disease. It is believed that epigenetic mechanisms mediate the interplay between environmental factors (e.g., diets) and the genome, leading to the development of various metabolic disorders including NAFLD.^[^ ^3^ ^]^ Therefore, we employed a comprehensive approach that integrates multi‐OMIC analysis and functional studies 1) to examine whether HFD, which has been believed to cause obesity and its associated disorders, modulates DNA methylome during the development of fatty liver and 2) to identify key gene(s) whose DNA methylation status is epigenetically altered by the HFD, thereby contributing to the development of hepatic steatosis. Using a genome‐wide RRBS analysis, we demonstrated that DNA methylome in the liver is dynamically altered by HFD feeding, which presents a stark contrast to the notion that DNA methylation is a relatively stable epigenetic mark compared to other epigenetic regulations such as histone methylation and acetylation. Notably, we discovered that the DNA methylation rate at the *Klb* promoter is enhanced by HFD feeding through RRBS analysis. Given the fact that the proximal promoter and 5’ region of *Klb* are enriched with CpG sites, we posited that *Klb* is subject to DNA methylation by HFD. Indeed, this was further confirmed by pyrosequencing analysis and luciferase assays. The enhanced DNA methylation at the *Klb* promoter may bear biological consequences, evident by the observation that *Klb* mRNA expression is down‐regulated by HFD feeding, which may increase DNMT1 expression at mRNA levels or via decreasing UBE3A‐mediated protein ubiquitination and degradation as a result of lower levels of UBE3A in the liver induced by HFD. Epigenetic programming of the *Klb* promoter by HFD down‐regulates *Klb* expression, leading to hepatic lipid accumulation in obesity.

Several lines of evidence support the importance of DNA methylation in the development of NAFLD. For instance, aberrant DNA methylation patterns have been associated with the development of NAFLD.^[^ ^21^ ^]^ This is congruent with the clinical studies also revealing altered CpG methylation on genes involved in the development of steatohepatitis, fibrosis, and carcinogenesis in patients with advanced NAFLD.^[^ ^22^ ^]^ The global change in DNA methylation might be partially explained by the observation on the increased expression of the DNA methylation enzyme DNMT1 in NAFLD.^[^ ^23^ ^]^ Despite the overexpression of DNMT1, which may potentially have a pleiotropic effect, the targets of the enzyme could still be pathway‐ and gene‐specific in a given tissue or a physiological scenario. For example, abnormal DNA methylation has been identified in genes responsible for specific pathways such as hepatic lipid metabolism. Compared with healthy subjects, patients with NAFLD exhibit increased CpG methylation in fatty acid oxidative genes and decreased methylation in fibrogenic genes, which may collectively contribute to the initiation and progression of NAFLD.^[^ ^24^ ^]^ Moreover, analysis of liver biopsy samples from a cohort of NAFLD patients showed that methylation levels at the promoter of the fatty acid oxidative gene PGC1*α* was negatively associated with its gene expression and positively associated with insulin resistance.^[^ ^25^ ^]^

We also determined the physiological impact of the altered methylome on hepatic steatosis. Since our RRBS data show that HFD tends to enhance DNA methylation in the genome, we reasoned that inhibiting DNA methylation through genetic approaches may correct the phenotype of hepatic steatosis. Indeed, we found that inhibiting DNA methylation by genetic deletion of *Dnmt1* or *Dnmt3a* dramatically ameliorates hepatic steatosis in diet‐induced obese mice. To examine the pathways mediating lipid metabolism, we found that *Dnmts’* deficiency suppresses the expression of genes involved in fatty acid oxidation and has some effects on the expression of genes involved in lipogenesis. This is associated with demethylation of the *Klb* promoter and upregulation of its mRNA expression, suggesting that *Klb* may serve as an epigenetic target that mediates the beneficial effect of *Dnmt* deficiency on hepatic steatosis. *Klb* is a coreceptor required for normal physiological functions of FGF15/19 and FGF21, which play an integral role in the regulation of key metabolic pathways.^[^ ^16^, ^17^ ^]^ For hepatic lipid metabolism, both FGF15/19 and FGF21 have been shown to antagonize hepatic steatosis via a coordinated control over stimulation of fatty acid oxidation and suppression of lipogenesis.^[^ ^16^, ^17^ ^]^ For instance, suppression of FGF21 in liver promotes hepatic steatosis via downregulation of hepatic fatty acid oxidation,^[^ ^26^ ^]^ whereas over‐expression of FGF21 in hepatocytes inhibits de novo lipogenesis via suppression of key lipogenic gene expression.^[^ ^27^ ^]^ Similarly, increasing FGF19, the human counterpart of FGF15, via pharmacological or genetic approaches, can promote fatty acid oxidation^[^ ^28^, ^29^ ^]^ while suppressing de novo lipogenesis in the liver.^[^ ^30^ ^]^ Since the main metabolic pathways that exhibits a dominant change in *Dnmt*‐deficient liver involve fatty acid oxidation and oxidative phosphorylation, it is conceivable that other genes with changes of DNA methylation at 5’‐end promoters in our RRBS data may also contribute the protective effects of *Dnmt1* or *Dnmt3a* deficiency on hepatic steatosis. Further studies are required to examine the methylation status on these candidate genes and unravel the molecular events underlying the metabolic dysfunctions in the liver.

Our snRNA‐seq analysis has shown a broad down‐regulation of genes involved in inflammatory pathways and fibrosis in Dnmt1‐deficient hepatocytes, suggesting a role of DNMT1 in hepatic inflammation. NASH is characterized by the presence of chronic inflammation and fibrosis in the setting of hepatic steatosis.^[^ ^31^ ^]^ The activation of inflammatory response in the liver is a major driving force of the pathological progression towards hepatic fibrogenesis,^[^ ^32^ ^]^ activation of which has also been discovered in our snRNA‐seq analysis. The key question centers on the identification of the mechanism responsible for the down‐regulated inflammation in *Dnmt1*‐deficient hepatocytes. Several plausible pathways have been advanced to unravel the mechanisms underlying the activation of inflammatory response in hepatic steatosis, including immune cell infiltration (e.g., macrophages), lipotoxicity, endoplasmic reticulum stress, oxidative stress, mitochondrial dysfunction, etc.^[^ ^31^ ^]^ However, future studies are warranted to uncover exactly how DNA methylation regulates the hepatic inflammatory program.

It is noteworthy that hepatic *Dnmt1* deficiency preferentially increases the proportion of periportal hepatocytes in the total number of liver cells. Because hepatocytes residing in the periportal zone harbor larger mitochondria with a strong ability to oxidize fatty acids due to their unique anatomic milieu exposed to the polarized blood flow rich in oxygen and nutrients,^[^ ^15^ ^]^ the disproportional increase of periportal hepatocytes is physiologically significant for Dnmt1‐deficient hepatocytes to maintain a healthy lipid homeostasis through mobilizing lipid oxidation against excess fatty acid influx during HFD feeding. In light of the periportal hepatocytes being adept at performing oxidative metabolism, our hierarchical cluster analysis showed a marked upregulation of genes responsible for oxidative phosphorylation and fatty acid oxidation in *Dnmt1*‐deficient hepatocytes. We posit that *Dnmt1* deficiency ameliorates HFD‐induced hepatic steatosis, at least in part, via increasing the proportion of the periportal hepatocytes. Future studies are required to address how hepatic *Dnmt1* deficiency promotes fatty acid oxidation in a zonation‐specific manner.

Along the course of our study, Kim et al showed that FGF19 ameliorates hepatic steatosis via DNMT3a‐mediated repression of lipogenesis.^[^ ^33^ ^]^ The role of DNMT3A in this study appears to be contradictory to the inhibitory effect of DNMT3A on Klb and fatty oxidation we observed in our study. The exact reason is not clear, but the two studies employed two different approaches to inhibit hepatic DNMT3A. While their study utilized AAV‐mediated shRNA to knock down *Dnmt3a* in the liver where other cells other than hepatocytes presumably underwent the knockdown regimen, we used a more hepatocyte‐specific Cre‐lox model where *Dnmt3a*‐floxed mice were injected with AAV virus carrying hepatocyte‐specific TBG Cre.^[^ ^9^, ^10^ ^]^ Further studies are required to explore this discrepancy.

Our data indicate that UBE3A regulates DNMT1 protein stability via ubiquitination‐mediated proteasomal degradation and that HFD feeding may enhance hepatic DNMT1 protein contents via inhibiting Ube3a expression. Although HFD feeding also appeared to augment DNMT3A protein contents in the liver, we did not observe any effect of UBE3A on DNMT3A protein stability. We posited that other ubiquitination pathways might mediate the effect of HFD on the DNMT3A protein stability. Future studies will be warranted to address this question. Along the course of our current study, there are two studies showing that HFD treatment may down‐regulate *Dnmt1* expression in the liver.^[^ ^34^, ^35^ ^]^ Remely et al reported that a 16‐week HFD feeding increased hepatic *Dnmt1* mRNA expression,^[^ ^34^ ^]^ while Li et al showed that treatment of C57BL/6 mice for 22 weeks promoted DNMT1 protein levels in the liver without change of *Dnmt1* mRNA.^[^ ^35^ ^]^ The exact reason for the discrepancy between their results and ours is not clear. The two studies employed a 22‐week and 16‐week HFD feeding regimen respectively, which are longer than that of our study. We speculate that the difference in HFD feeding duration may cause the difference in the effect of HFD on hepatic DNMTs’ expression. In addition, the fat content in the HFD used by Remely's study is slightly different from ours, which may have an impact on the expression of *Dnmt1* in the liver, since different HFDs with varied fat contents may cause the variations of hepatic steatosis.^[^ ^36^, ^37^ ^]^

Apart from the observations that different fat contents in HFDs may cause the varying degrees of hepatic steatosis and subsequently the DNA methylome, the mouse strain, and sex may also have impacts on the hepatic DNA methylation.^[^ ^38^ ^]^ For example, mouse strains and sex that are prone to develop NAFLD are associated with a genome‐wide DNA hypermethylation.^[^ ^38^ ^]^ Along this line, the discovery of our study that employed the male C57BL/6J mice may not be extrapolated to other mouse strains, female mice, and humans.

In summary, our study demonstrates that the DNA methylome is under dynamic regulation during the development of diet‐induced hepatic steatosis in male C57BL/6J mice, in which *Klb* may be an epigenetically regulated target of DNA methylation by HFD. We conclude that up‐regulation of DNA methyltransferases by nutrient‐rich diets may result in hypermethylation of the *Klb* promoter and subsequent down‐regulation of *Klb* expression, thereby impairing fatty acid oxidation; this may in turn contribute to the development of NAFLD through the suppression of oxidative phosphorylation and fatty acid oxidation in male C57BL/6J mice.

## 4 Experimental Section

### Mice

All animal procedures were conducted in accordance with the Institutional Animal Care and Use Committee guideline at Georgia State University (A22004). For diet‐induced obesity studies, 6‐week‐old male C57BL/6J mice were purchased from the Jackson Laboratory (Bar Harbor, ME) and were fed either a LFD (D12450B, 10% kcal from fat, Research Diets Inc., New Brunswick, NJ) or HFD (D12492, 60% calorie from fat, Research Diets Inc.) up to 24 weeks. At the end of the study, liver and various fat pads were dissected, weighed, and snap‐frozen in liquid nitrogen for further analyses.

We have generated mice with liver‐specific deletion of *Dnmt1*(LD1KO), *Dnmt3a* (LD3aKO), or *Tet2* (LT2KO) by intravenously injecting AAV8‐human thyroid hormone binding globulin (TBG)‐Cre virus (produced by UPenn Viral Vector Core)^[^ ^9^, ^10^ ^]^ into *Dnmt1*‐floxed mice from the NIH‐supported Mutant Mouse Regional Resource Centers (MMRRC, No. 01 4114); *Dnmt3a*‐floxed mice (No. 02 9885, MMRRC), or Tet2‐floxed mice (No. 01 7573; the Jackson Laborary, Bar Harbor, ME). The *Dnmt1*‐floxed mouse was created by inserting two loxP sites flanking exons 4 and 5, which causes frameshift and lacks the motifs for the catalytic domain.^[^ ^39^ ^]^ The *Dnmt3a*‐floxed mouse was created by inserting two loxP sites flanking exon 19, which encodes the catalytic motif.^[^ ^40^ ^]^ The *Tet2*‐floxed mouse was generated with the third exon flanked by two loxP sites.^[^ ^41^ ^]^ All three mouse models have been backcrossed to B6 background for multiple generations in our lab. Although both LD1KO and LD3aKO lines (and their respective fl/fl controls) have been backcrossed to C57/BL6J background for more than 8 generations, the two lines may still have subtle differences in genetic background, which may affect their degrees of hepatic steatosis and time required for such development in responses to the same HFD feeding. During the HFD feeding study, we therefore monitored major metabolic phenotypes including body weight and blood lipid profile to ensure a comparability of the metabolic phenotypes between LD1KO and LD3aKO lines. We observed a slightly slower development of body weight gain and increased blood TG levels in LD3aKO mice. We therefore put LD1KO mice on the HFD for 10 weeks while LD3aKO mice for 12 weeks.

For the models with liver *Tet1*, *Tet2*, or *Tet3* knockdown, 7‐week‐old male C57BL/6J mice were intravenously injected with AAV8 *Tet1* or *Tet2* or *Tet3* shRNA purchased from Addgene (Tet1 shRNA 85 742, Tet2 shRNA 86 743, Tet3 shRNA 85 740, and Control 85 741). After 1 week of recovery, mice were fed HFD for 5 weeks.

### Specific Demethylation of the Klb Promoter

The specific demethylation of the Klb promoter was conducted as we previously described.^[^ ^42^ ^]^ The lentiviral vector expressing dCas9‐Tet1 was purchased from Addgene (No. 84 475). The guide RNA sequences targeting DNA methylation at the *Klb* promoter were designed with the online software at the GT‐Scan website (http://gt‐scan.braembl.org.au/gt‐scan). The targeting or non‐targeting oligos were subcloned into the AarI sites of the pgRNA lentiviral vector (No. 44 248, Addgene). The targeting guide RNA sequences for the Klb promoter were: forward, 5′‐ttgg CCGTGCACTTCTGGACTCGCTGG ‐3′, reverse, 5’ ‐aaacCCAGCGAGTCCAGAAGTGCACGG ‐3′. The sequences for non‐targeting gRNA were: forward, 5′‐ttggCCCCCGGGGGAAAAATTTTT; reverse, 5′‐ aaacAAAAATTTTTCCCCCGGGGG‐3. Lentiviruses expressing dCas9‐TET1 or gRNA‐mCherry (1  ×  109 IFU/ml) were produced by Vigene Biosciences, Inc., and were intravenously injected into 7‐week‐old male C57BL/6J mice.

### RRBS Analysis

RRBS was conducted as we described.^[^ ^42^ ^]^ Briefly, the genomic DNAs from mouse liver were extracted using a phenol chloroform extraction method. Five liver DNA samples were pooled for each group (high‐fat diet vs low fat diet) and were sent to Beijing Genomics Institute (BGI) (Shenzhen, China) that carried out the RRBS process including methylation‐insensitive restriction enzyme digestion, ligation to adaptors, fragment size selection, bisulfite conversion, PCR amplification, library construction, and sequencing. Bioinformatic analysis of RRBS data including differentially methylated regions (DMRs), methylation rate, and pathway analysis were provided by the BGI Bioinformatics Center or conducted by our co‐author Dr. Shi using the bioinformatics analysis pipelines as described.^[^ ^43^ ^]^ The methylation level at each CpG site was determined by the ratio of the number of sequences containing methylated CpGs divided by the total number of sequences. For the comparison of DNA methylation rates between the HFD‐fed and LFD‐fed mice, density plots tagged around the DNA structures including transcription start and termination sites, exon‐intron boundaries, CpG islands, and repeat elements were mapped to the University of California Santa Cruz (UCSC) Genome Browser on Mouse (NCBI37/mm9) Assembly for methylated gene alignment as described.^[^ ^43^ ^]^

### RNA‐Sequencing Analysis

The total RNA extracted from liver was sent to the Beijing Genomics Institute (BGI, Shenzhen, China) that conducted the deep sequencing process.^[^ ^42^, ^44^ ^]^ Clean reads were aligned to the reference genome (UCSC mm9) using SOAP2. Peak reads mapped to the annotated genes were counted using featureCounts at the Galaxy server (version 1.6.3.). Gene sets were considered to have a difference in expression with a fold change of 1.5 or greater.

### ATAC‐seq

The ATAC‐seq analysis was conducted as we previously described.^[^ ^42^ ^]^ Briefly, 30 mg of liver samples were homogenized in a dounce homogenizer and then centrifuged in a density solution containing iodixanol to isolate nuclei. The purified nuclei were then incubated with Nextera Tn5 transposase (Illumina) at 37 °C for one hour. The DNAs in the transposition mixture were purified and PCR amplified with NEBNext 2X MasterMix and Nextera Index primers to construct the ATAC libraries, which were further size‐selected by removing large DNA fragments and were submitted to Novogene (Durham, NC) that carried out the deep sequencing. The bioinformatic analysis of the ATAC‐seq data was conducted on the Galaxy server (version 1.6.3.) as we described.^[^ ^42^ ^]^

### snRNA‐seq)

The liver nuclei isolated as described above were sent to the Georgia Tech genomic core facility that carried out snRNA‐seq using a protocol described previously.^[^ ^45^ ^]^ Briefly, purified nuclei were processed for the construction of snRNA‐seq libraries using the Chromium Controller (10X Genomics, Inc, Pleasanton, CA, 94 588), which were sequenced using Illumina NextSeq500 sequencer with each nucleus being tagged with a 16 bp barcode and yielding 80k reads. The raw counts of the fastq files were analyzed for read alignment using the 10Xgenomic Cell Ranger online software (https://www.10xgenomics.com/products/cloud‐analysis). The outputs from the cell ranger analysis were further processed by using R package Seurat 4.^[^ ^46^ ^]^ Nuclei expressing > 7000 genes and nuclei with a higher percentage of mitochondrial genes were filtered out in the Seurat software. The top 2000 differentially expressed genes were selected by the vst method in Seurat 4. Unsupervised clustering was applied after aligning the 50 dimensions resulting from PCA with a resolution of 1.5. A higher resolution was applied for sub‐clustering the hepatocyte subpopulations. The UMAP plots, bar plots, violin plot, volcano plot, and heatmaps were generated by R and GraphPad.

### Antibodies

The antibodies used in immunoblotting, chromatin immunoprecipitation (ChIP) assays, and immunoprecipitation included KLB (AF2619, R&D Systems), DNMT1 (IMG‐261A, IMGENEX), DNMT3a (IMG‐268A, IMGENEX), GAPDH (6C5) (sc‐32233, Santa Cruz)), E6AP (sc‐166689, Santa Cruz), and UB (sc‐8017, Santa Cruz).

### Cell Culture, Ubiquitin‐Protein Ligase E3A (Ube3a) Knockdown or Overexpression

HEK293 cells obtained from American Type Culture Collection (ATCC; Manassas, VA) were cultured in Dulbecco's Modification of Eagle's Medium (DMEM) containing 10% fetal bovine serum (FBS) and 1% Penicillin/Streptomycin in an incubator with 5% CO2 at 37 °C. DMEM, Opti‐MEM Medium, FBS, and Penicillin/Streptomycin were purchased from Life Technologies (Grand Island, NY). The Ube3a knockdown or overexpression stable cell line was established as we previously described.^[^ ^47^ ^]^ For *Ube3a* overexpression, HEK293 cells were transfected with pLenti6‐Myc‐Ube3a lentivirus and selected with 1 µg mL^−1^ puromycin. For *Ube3a* knockdown, HEK293 cells were transfected by lentiviral GPIZ Ube3a shRNA from GE Dharmacon (Lafayette, CO).

### Primary Hepatocyte Isolation, Oxygen Consumption Rate (OCR) and Hormonal Treatment

Mice were perfused via portal veins first with the perfusion medium (GIBCO #17701‐038, ThermoFisher Scientific, Waltham, MA) and then with the liver digest medium (GIBCO #17703‐034, ThermoFisher Scientific). The perfused liver was then dissected and washed in a cold washing medium (WEM, GIBCO #A1217601, ThermoFisher Scientific) and was minced and filtered through a 100 µm nylon filter. The cells were centrifuged in a cold percoll density solution for the pure hepatocytes, which were subsequently cultured in growth medium (WEM with 10% serum).

OCR in primary hepatocytes was measured using a XF 96 Extracellular Flux Analyzer (Agilent, Santa Clara, CA) as we described.^[^ ^42^ ^]^ Briefly, the assays began with a basal respiration measurement, and then a series of reagents including oligomycin, FCCP, rotenone, and antimycin A were added to measure ATP‐linked, maximum, and nonmitochondrial respiration rates.

Primary hepatocytes were also isolated from *Dnmt1*‐ or *Dnmt3a*‐floxed mice as described above and were infected with AAV8‐TBG‐Cre AAV or control virus to generate *Dnmt1* or *Dnmt3a*‐deficient hepatocytes and their respective control hepatocytes. The primary hepatocytes were then treated with FGF15 (20 nM; Cat. Ab206457; Boston, MA) or FGF21 (20 nM; Cat. 2539FG025, R&D Systems, Minneapolis, NM) for 4 h before harvested for RNA extraction.

### Total RNA Extraction and Quantitative RT‐PCR

Total RNA was extracted from liver using the Tri‐Reagent kit (Molecular Research Center, Cincinnati, OH) as it was previously described.^[^ ^42^ ^]^ The expression of genes of interest was quantitated by quantitative RT‐PCR (ABI Universal PCR Master Mix, Applied Biosystems, Foster City, CA) using an Applied Biosystems QuantStudio 3 real‐time PCR system (ThermoFisher Scientific).^[^ ^42^ ^]^ The primer and probe pairs used in the assays were purchased from Applied Biosystems.

### Immunoblotting (IB)

IB was performed as we previously described.^[^ ^42^, ^48^ ^]^ Briefly, liver samples were homogenized in a modified radioimmunoprecipitation assay (RIPA) lysis buffer. The homogenates were separated by SDS‐PAGE. Proteins on the gels were transferred to nitrocellulose membrane (Bio‐Rad, Hercules, CA), followed by blocking, washing, and incubating with various primary antibodies and the Alexa Fluor 680‐conjugated secondary antibody (Life Science Techenologies). The blots were developed with a Li‐COR Imager System (Li‐COR Biosciences, Lincoln, NE).

### UBE3A‐mediated Ubiquitination and Degradation of DNMT1 Protein

HEK293 cells expressing shUbe3a, shUbe3a + Ube3a cDNA, or Ube3a cDNA were treated with 10 µM MG132 (American Peptide, Sunnyvale, CA) for 90 min after 72‐h posttransfection. The cells were washed twice with PBS, lyzed by RIPA buffer, and centrifuged to extract the supernatant, which was precleared by the control IgG and protein A/G PLUS‐agarose. The cleared cell lysates containing 2 mg total protein was incubated with the DNMT1 primary antibody (IMG‐261A, IMGENEX) and protein A/G PLUS‐Agarose at 4°C overnight, washed with PBS, boiled and loaded in SDS‐PAGE and immunoblotted with the ubiquitin antibody (sc‐8017, Santa Cruz).

### Cloning of the Mouse Klb Promoter and the Luciferase Reporter Assays

A mouse 700 bp *Klb* promoter was PCR amplified from a bacterial artificial chromosome clone using the following primers: *Klb* forward: 5’‐AAAGTTTAAAATATTTAGAAAGGTTT‐3’; *Klb* reverse: 5’‐AAAACCTATAATTATAAAACCCTATCAA‐3’. The PCR products were cut with XhoI/HindIII and then inserted into pGL3.1‐Basic at XhoI/HindIII sites to generate pGL3.1‐Klb. The constructs were confirmed by sequencing.

The luciferase reporter assays to assess methylated promoter activity were conducted as we previously described.^[^ ^49^ ^]^ The unmethylated *Klb* promoter was obtained by transforming the luciferase reporter constructs into the *dam‐/dcm‐ E. coli* strain (New England Biolabs, Ipswich, MA), while the fully methylated Klb promoter was obtained by incubating the reporter constructs with the SssI methylase in the presence of S‐adenosylmethionine (New England Biolabs). The unmethylated or fully methylated *Klb* promoter reporter constructs were then transfected into Hepa 1–6 cells and the luciferase activity was measured using a Dual Luciferase Reporter Assay System (Promega, Madison, WI).

### Bisulfite Conversion and Pyrosequencing of the Klb Promoter

Pyrosequencing analysis of the CpG sites at the Klb promoter was conducted as we previously described.^[^ ^49^ ^]^ A total of 1 µg of genomic DNA extracted from the liver samples using the phenol/chloroform method was converted using the EpiTect Bisulfite kit (Qiagen, Valencia, CA) and purified. The bisulfite‐converted DNAs were purified and the fragments covering putative CpG sites at the *Klb* promoter were PCR‐amplified and sequenced commercially by EpigenDx (Hopkinton, MA). The pyrosequencing primers for the *Klb* promoter were designed using PyroMark Assay Design 2.0 software (Qiagen). The pyrosequencing data were analyzed using Pyro‐Q‐CpG software (version 1.0.9) (Qiagen).

### Chromatin Immunoprecipitation (ChIP) Assays

ChIP assays were performed using a ChIP assay kit (Upstate, Lake Placid, NY) as we previously described.^[^ ^50^ ^]^ Briefly, liver tissues were minced into small pieces and fixed with 1% of formaldehyde and were then homogenized in a glass dounce homogenizer to isolate nuclei, which were resuspended in nuclei lysis buffer and sonicated to shear genomic DNA to an average fragment length of 200–1,000 bp with a Diagenode Bioruptor (Diagenode, Denville, NJ). The lysates were centrifuged to obtain supernatants, which were used for immunoprecipitation. The DNAs extracted from the lysates were used for quantitative PCR analysis using the SYBR Green approach (Applied Biosystems). The sequences of primers for the *Klb* promoter regions were as follows: forward: 5’‐ATGAAATTACCCGTCAAACTC‐3’; *Klb* reverse: 5’‐CAATGATTAGCCTGGATCGG‐3’.

### Statistics

One‐way analysis of variance (ANOVA) and least‐significant‐difference test or T test were performed to evaluate statistical significance using GraphPad Prism version 5.0. Statistical significance was considered at *p <* 0.05. All data are shown as mean ± standard error (SEM).

### Study Approval

All animal studies were approved by the Institutional Animal Care and Use Committee at Georgia State University (A22004).

## Conflict of Interest

The authors declare no conflict of interest.

## Author Contributions

S.W., L.Z., Y.T.Y., RL, X.C. and JJ performed most experiments and analyzed the data. HDS performed RRBS data analysis. S.W., X.C., L.Y., H.D.S., WC, and JH contributed to the snRNA‐seq analysis. RL and JY contributed to the ubiquitination assays. JY and LY contributed to the data interpretation and discussion. J.Y. and L.Y. reviewed and edited the manuscript. H.S., B.X., and L.Y. conceived the hypothesis, designed the study, and analyzed the data. H.S., S.W., and B.X. wrote the manuscript.

## Supporting information

Supporting Information

## Data Availability

The data that support the findings of this study are available from the corresponding author upon reasonable request.
