# Supplementary material for: Epigenetic Regulation of Hepatic Lipid Metabolism by DNA Methylation
Source: Adv Sci (Weinh). 2023 Jun 6;10(20):2206068. doi: 10.1002/advs.202206068 (PMC10369300; doi:10.1002/advs.202206068)
Supplement: Supplementary file 1 — Supporting Information [file ADVS-10-2206068-s001.pdf]

ADVANCED SCIENCE
Open Access

Supporting Information

for *Adv. Sci.*, DOI 10.1002/advs.202206068

Epigenetic Regulation of Hepatic Lipid Metabolism by DNA Methylation

Shirong Wang, Lin Zha, Xin Cui, Yu-Te Yeh, Ruochuan Liu, Jia Jing, Huidong Shi, Weiping Chen, John Hanover, Jun Yin, Liqing Yu, Bingzhong Xue* and Hang Shi**

Supplemental figure legends

Supplemental figure 1: Induction of hepatic steatosis in male C57BL/6J mice by 12-week HFD feeding. (A) Liver TG contents. (B) Oil red O staining of the liver. Male C57BL/6J mice were fed either HFD or LFD for 12 weeks. All data are expressed as mean±SEM. n=7-8; *p<0.05 vs. LFD.

Supplemental figure 2: *Dnmt3b* mRNA, protein and chromatin accessibility in the liver of HFD-fed mice. (A) Quantitative RT-PCR analysis of *Dnmt3b* mRNA in the liver of mice with either HFD or LFD feeding (n=6-8). (B) Immunoblotting analysis of DNMT3B protein (n=4). (C) The association of the peaks of the chromatin accessibility at the *Dnmt3b* promoter analyzed by ATAC-seq and the reads of the *Dnmt3b* mRNA expression analyzed by RNA-seq. Male C57BL/6J mice were fed either HFD or LFD. All data are expressed as mean±SEM; *p<0.05 vs. LFD.

Supplemental figure 3: Generation of LD1KO mice. (A) Quantitative RT-PCR analysis of *Dnmt1* mRNA and immunoblots of DNMT1 protein in LD1KO mice and control fl/fl mice respectively. (B) Quantitative RT-PCR analysis of *Dnmt3a* mRNA and immunoblots of DNMT3A protein in LD3aKO mice and control fl/fl mice respectively. All data are expressed as mean±SEM. n=6-8; *p<0.05 vs. fl/fl.

Supplemental figure 4: Knockout efficiency of *Dnmt1* and *Dnmt3a* in primary hepatocytes isolated from LD1KO or LD3aKO mice. (A) Quantitative RT-PCR analysis of *Dnmt1* mRNA and immunoblots of DNMT1 protein in primary hepatocytes of LD1KO and control fl/fl mice respectively. (B) Quantitative RT-PCR analysis of *Dnmt3a* mRNA and immunoblots of DNMT3A protein in primary hepatocytes of LD3aKO and control fl/fl mice respectively. All data are expressed as mean±SEM. n=3-8; *p<0.05 vs. fl/fl.

Supplemental figure 5: Metabolic characterization of LD1KO mice. (A) Body weight. (B) Fat

pad weight. Epididymal fat: Epi; Retroperitoneal fat: RP; Brown adipose tissue: BAT; Subcutaneous fat: SQ. (C) Circulating lipid profile including triglyceride (TG), total cholesterol (TC) and free cholesterol (FC). All data are expressed as mean±SEM. n=6-8; *p<0.05 vs. fl/fl.

Supplemental figure 6: Metabolic characterization of LD3aKO mice. (A) Body weight. (B) Fat pad weight. Epididymal fat: Epi; Brown adipose tissue: BAT; Subcutaneous fat: SQ. (C) Circulating lipid profile including TG, TC and FC. All data are expressed as mean±SEM. n=6-8; *p<0.05 vs. fl/fl.

Supplemental figure 7: Quantitative RT- PCR analysis of *Tets'* expression in the liver of mice fed with HFD or LFD. All data are expressed as mean±SEM; n=8; *p<0.05 vs. control.

Supplemental figure 8: Chromatin accessibility and gene expression of *Tets* in the liver of HFD-fed mice. The association of the peaks of the chromatin accessibility at the *Tet1*, *Tet2*, or *Tet3* promoter analyzed by ATAC-seq, respectively (upper panels) and the reads of the *Tet1*, *Tet2*, or *Tet3* mRNA expression analyzed by RNA-seq, respectively (lower panels).

Supplemental figure 9: Knocking down *Tets* with AAV shRNA in the liver. (A) Quantitative RT-PCR analysis of Tets knockdown (KD) efficiency in the liver of mice infected with AAV *Tet* shRNAs or control scramble RNA virus. (B) Body weight and liver weight of mice infected with AAV Tet shRNAs or control virus. (C) Liver TG contents. (D) Representative histology of the liver of mice infected with AAV *Tet* shRNAs. All data are expressed as mean±SEM; n=4; *p<0.05 vs. control.

Supplemental figure 10: Metabolic characterization of LT2KO mice. (A) Body weight. (B) Fat pad weight. Epididymal fat: Epi; Brown adipose tissue: BAT; Subcutaneous fat: SQ. (C) Circulating TG, TC and FC. All data are expressed as mean±SEM. n=5; *p<0.05 vs. fl/fl.

Supplemental figure 11: (A) Heatmap of gene expression of phagosome formation in the

hepatocytes of LD1KO mice and fl/fl mice. (B) Heatmap of gene expression of fibrosis signaling in the hepatocytes of LD1KO mice and fl/fl mice.

Supplemental figure 12: Quantitative RT-PCR analysis of lipogenic gene expression in the liver of LD1KO mice (A), LD3aKO mice (B) and LT2KO mice (C), respectively. All data are expressed as mean±SEM. n=5; *p<0.05 vs. fl/fl.

Supplemental figure 13: Quantitative RT-PCR analysis of the expression of genes involved in metabolism identified by RRBS analysis**.** All data are expressed as mean±SEM; *p<0.05 vs. LFD.

Supplemental figure 14. A schematic illustration of the Klb promoter and 5' region. TATA box and exon 1 are indicated. The CpG sites measured by pyrosequencing analysis are indicated as upward vertical lines with solid circles. Position 1-10 are located downstream of the TATA box at the promoter region, while position 13-24 are located at the beginning of the first exon.

Supplemental figure 15: Signal network analysis using the differentially expressed genes of the Dnmt1-deficient hepatocytes from the snRNA-seq dataset reveals KLB as a metabolic nexus.

Supplemental figure 16: Metabolic characterization of mice infected with dCas9-Tet1 and sgRNA virus or dCas9-Tet1 and scramble RNA lentivirus. (A) Body weight. (B) Fat pad weight. (C) Quantitative RT-PCR analysis of lipogenic gene expression. (D) Quantitative RT-PCR analysis of gene expression involved in bile acid synthesis. (E) Quantitative RT-PCR analysis of *Fgf21* gene expression. All data are expressed as mean±SEM. n=7-10; *p<0.05 vs. control.

Supplemental figure 17: Metabolic characterization of mice infected with dCas9-Dnmt3a and sgRNA virus or dCas9-Dnmt3a and scramble RNA lentivirus. (A) Body weight. (B) Pyrosequencing analysis of DNA methylation rate in the CpG sites of the *Klb* promoter. (C)

Quantitative RT-PCR analysis of *Klb* mRNA expression. (D) Liver weight. (E) Liver TG contents. (F) Circulating TG. (G) Histology of the liver. All data are expressed as mean±SEM. n=5-10; *p<0.05 vs. control.

Supplemental figure 1

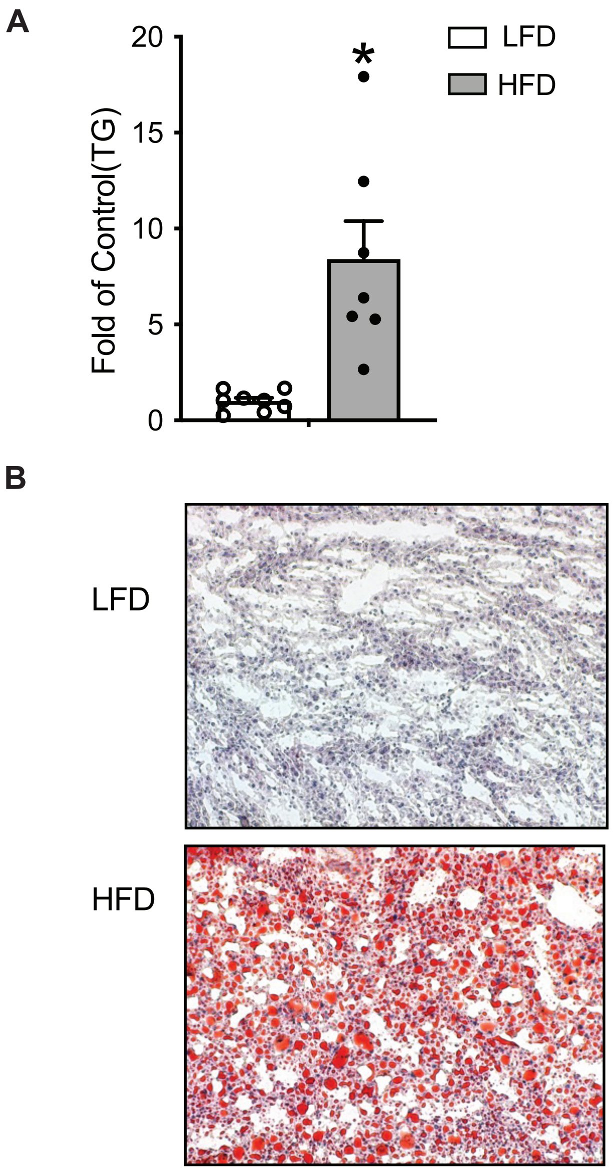

Supplemental figure 2

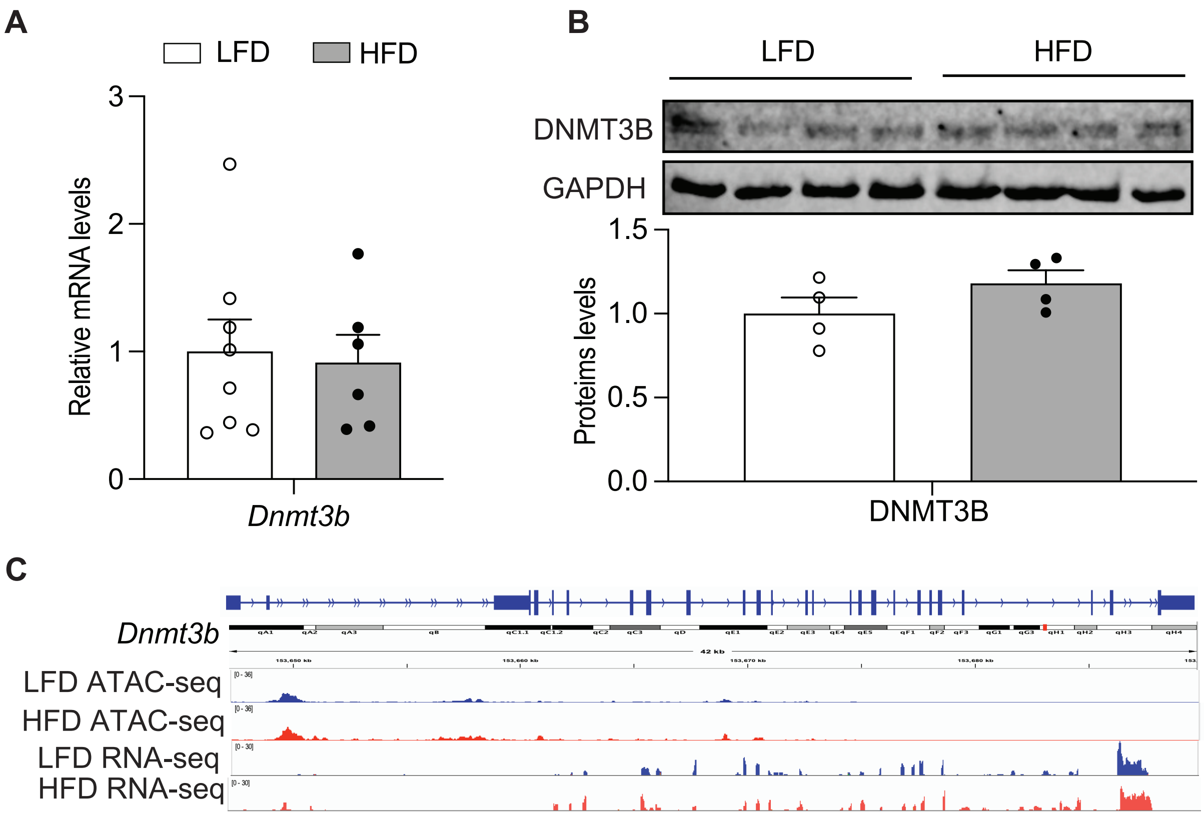

Supplemental figure 3

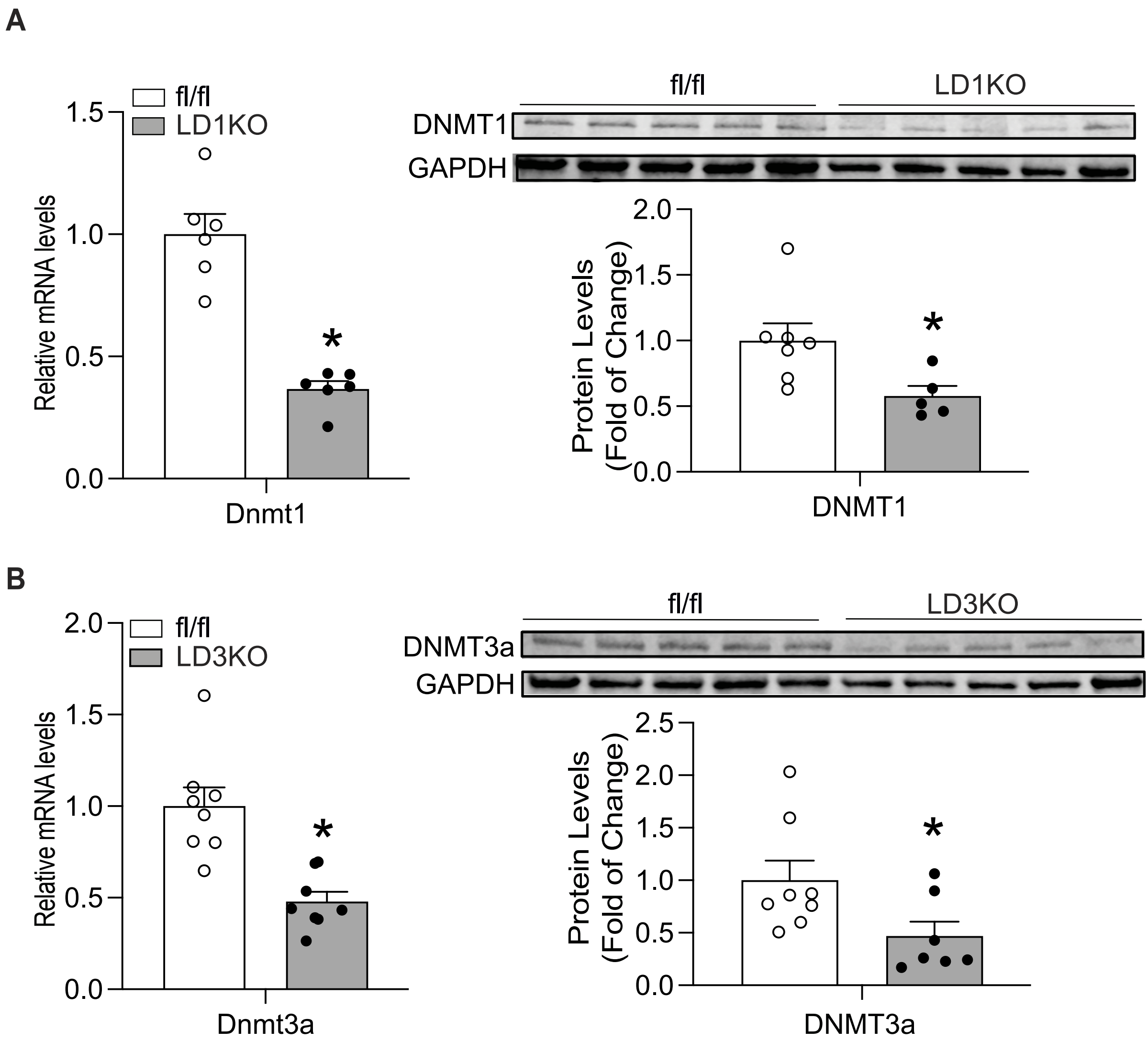

Supplemental figure 4

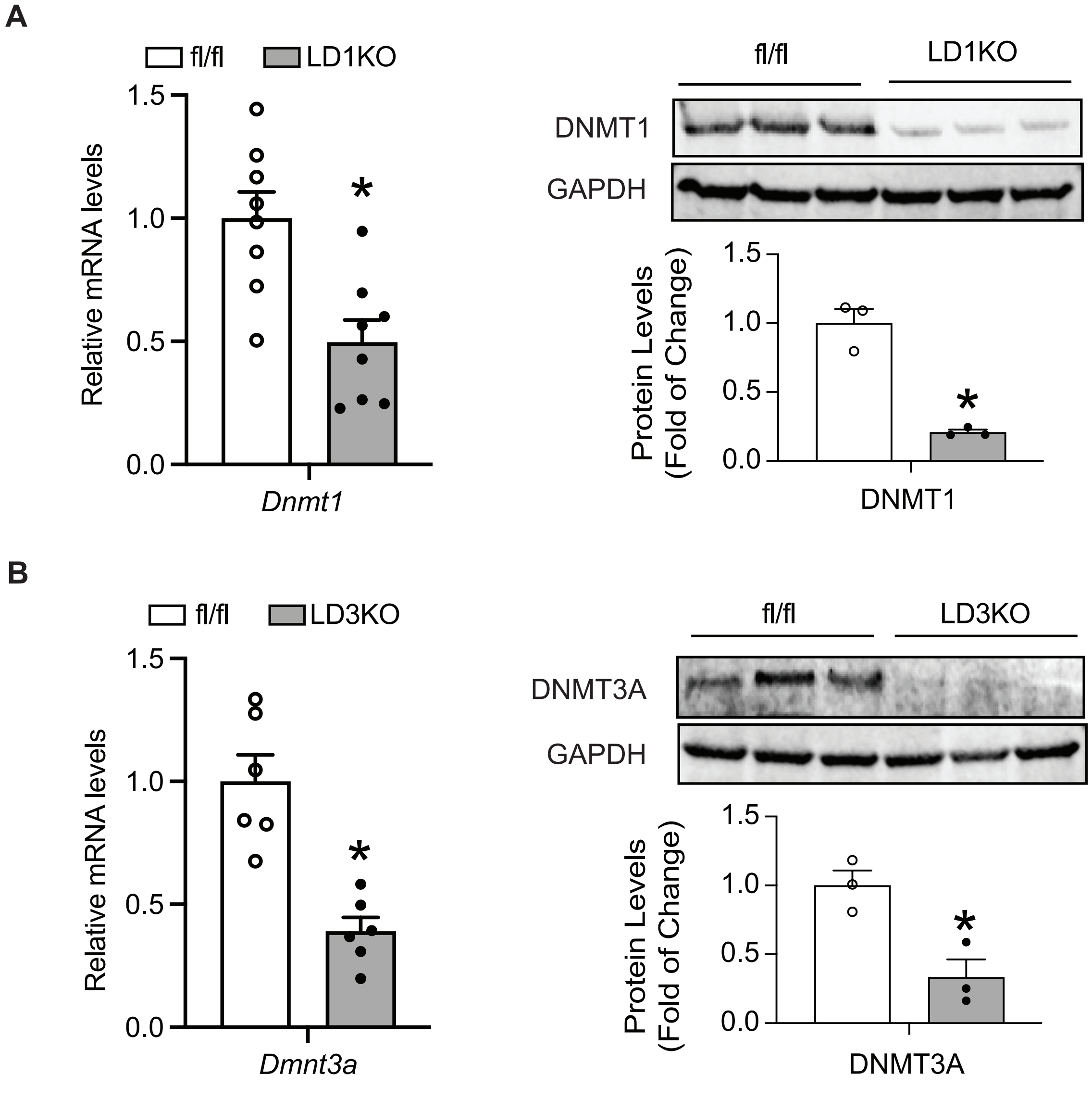

Supplemental figure 5

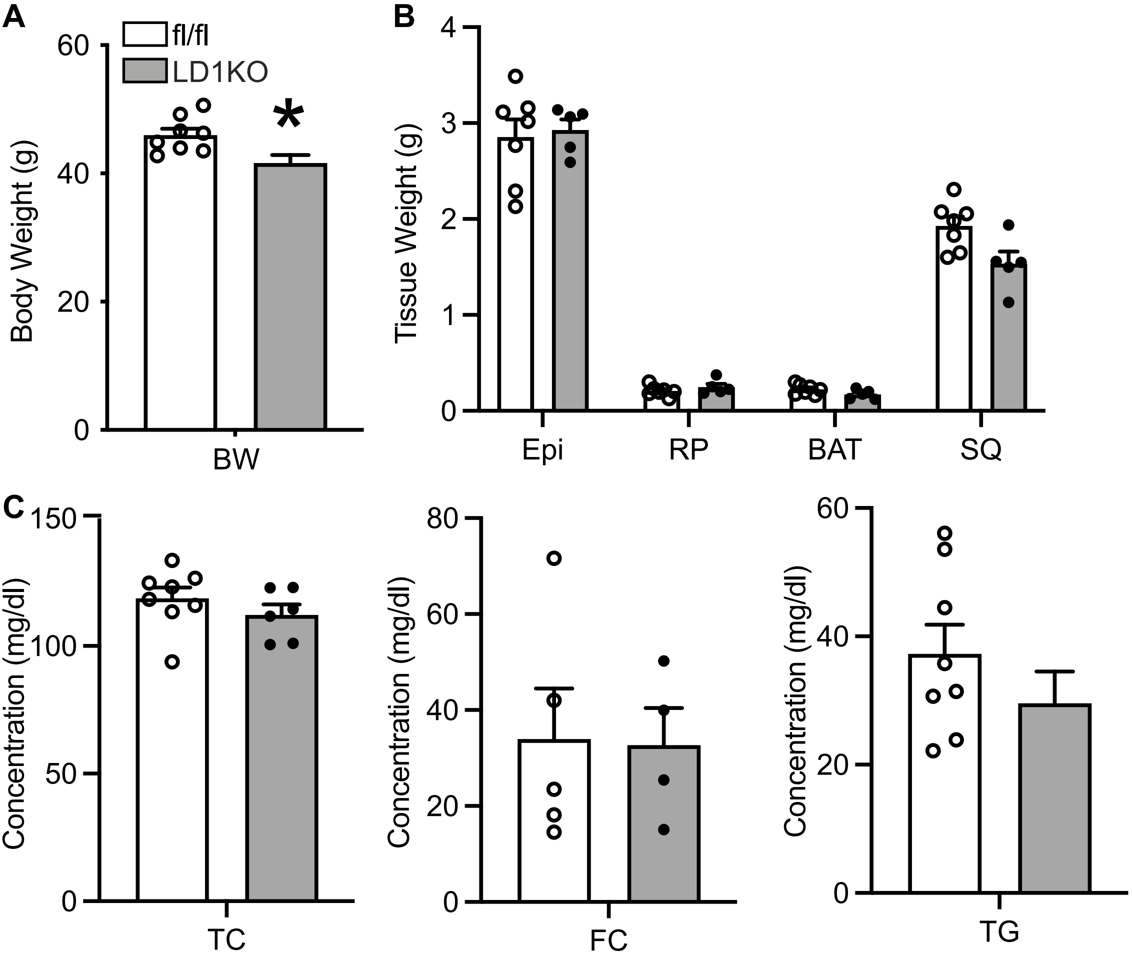

Supplemental figure 6

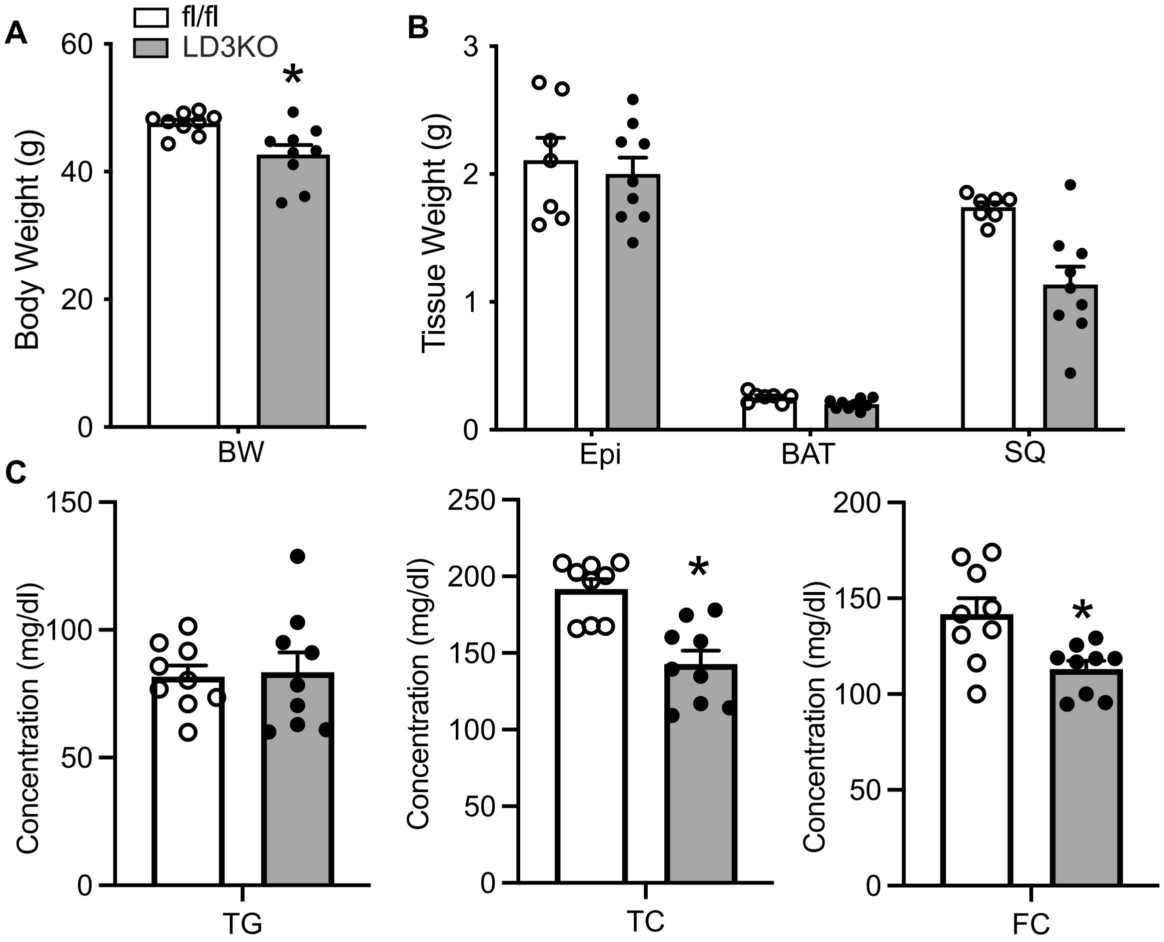

Supplemental figure 7

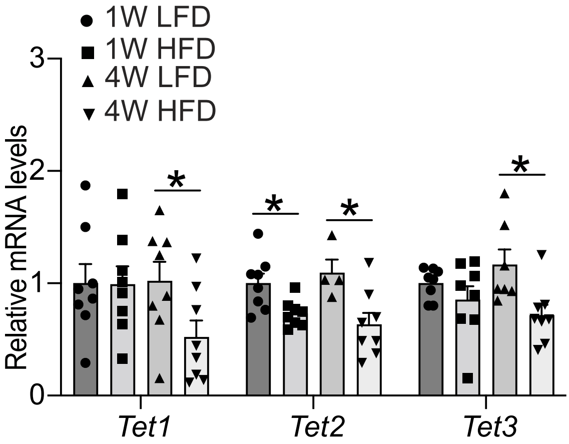

Supplemental figure 8

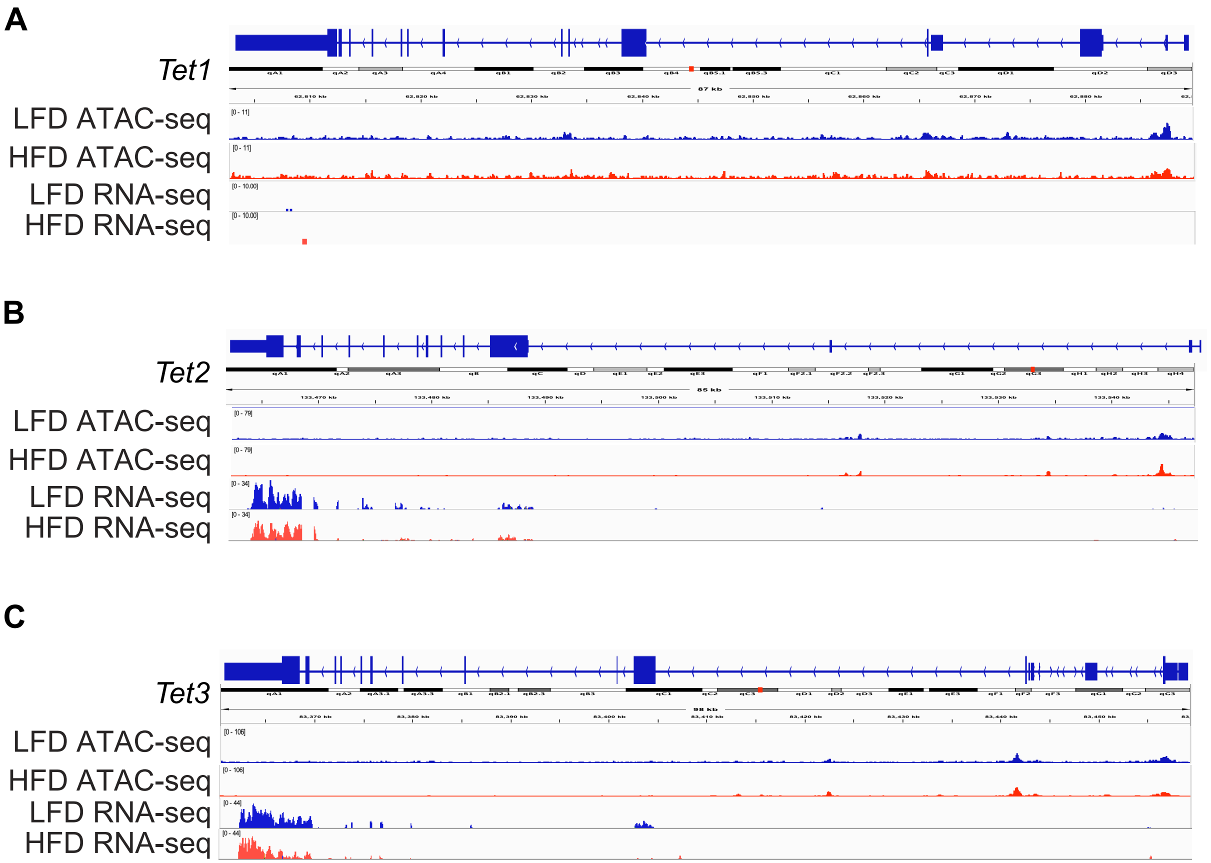

Supplemental figure 9

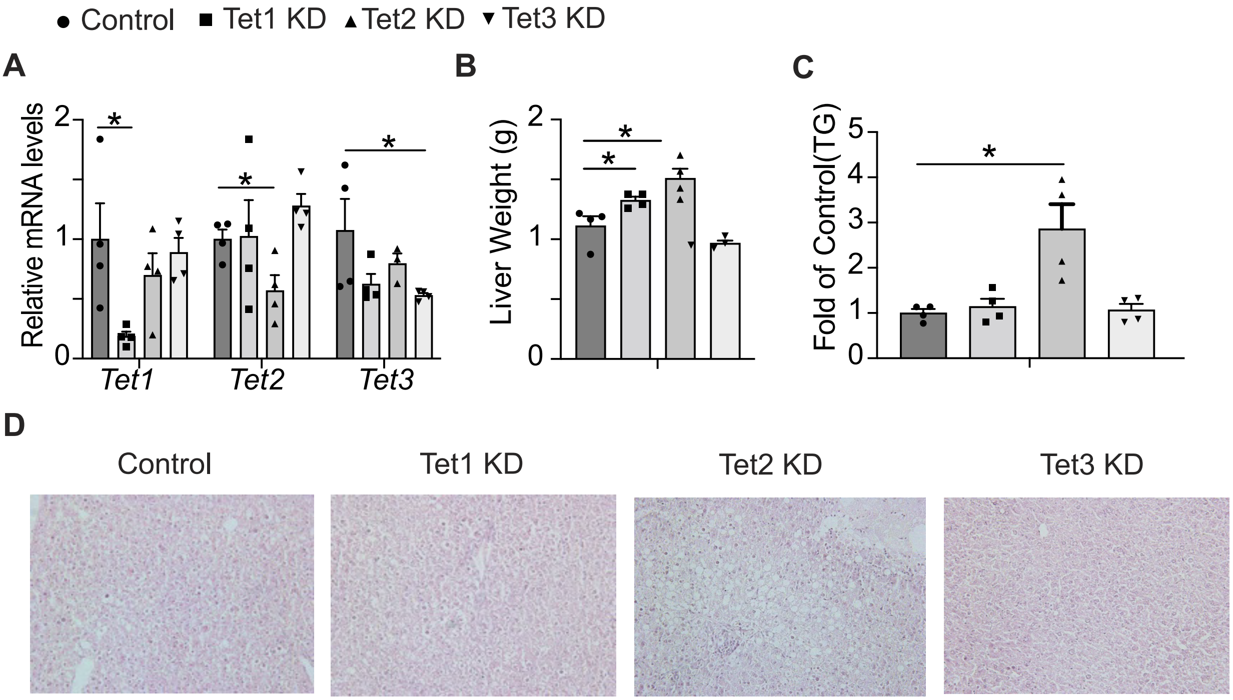

Supplemental figure 10

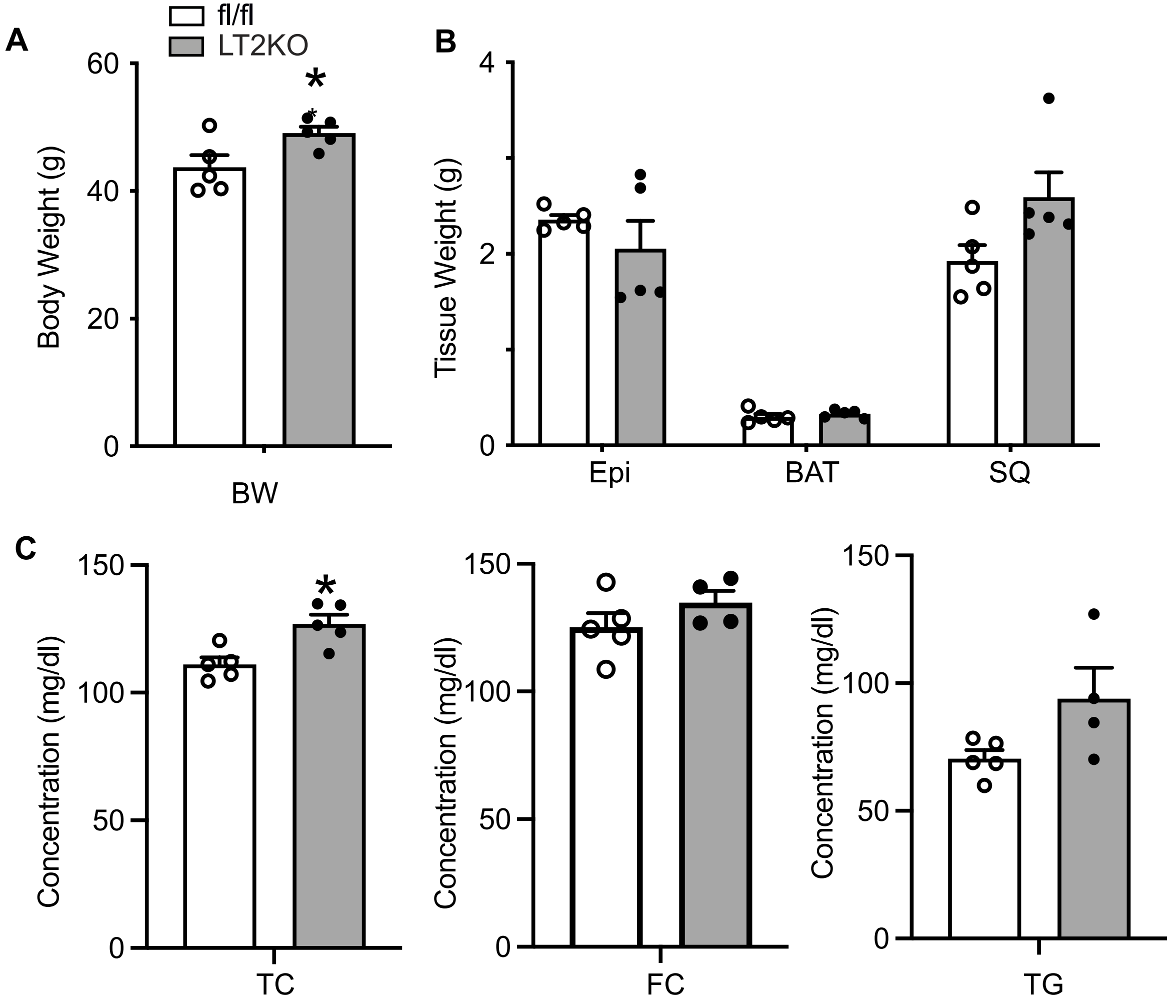

Supplemental figure 11

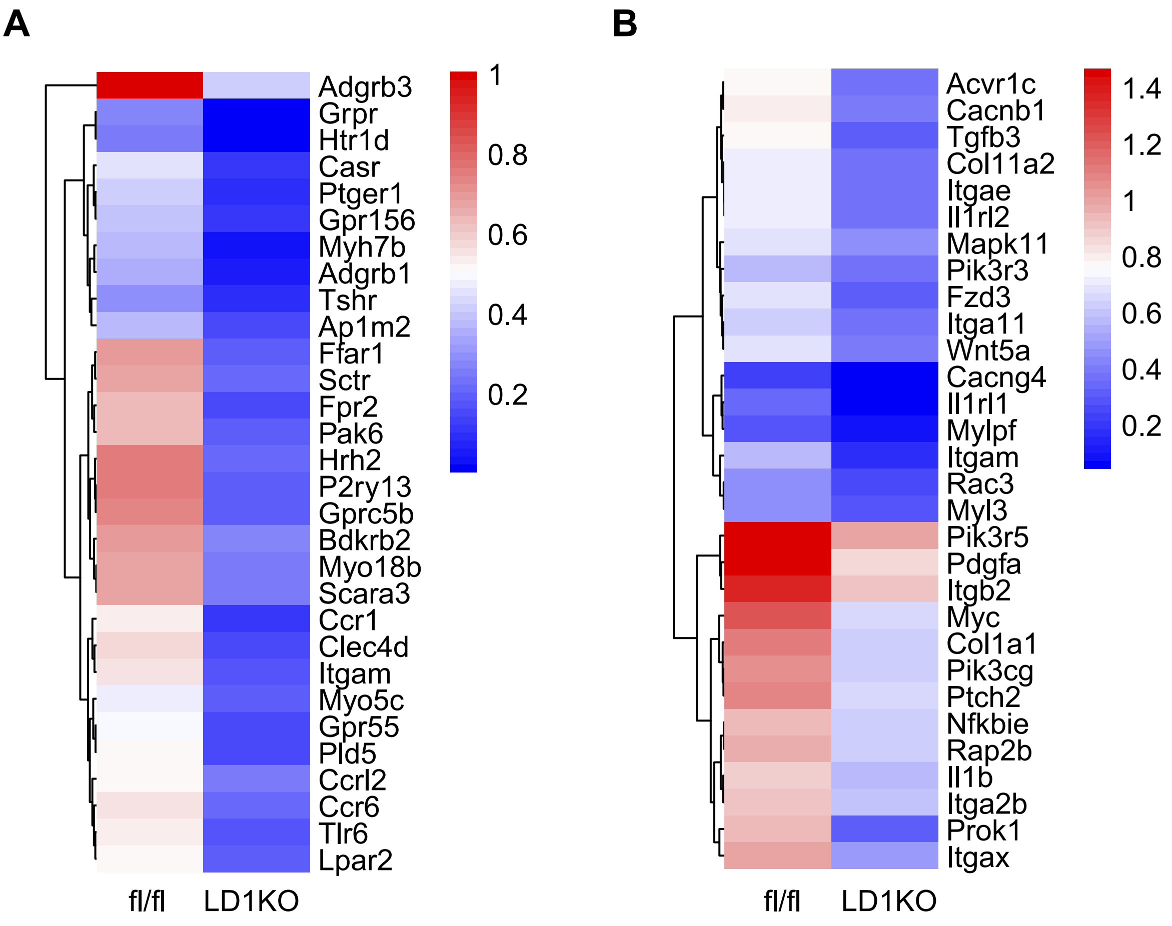

Supplemental figure 12

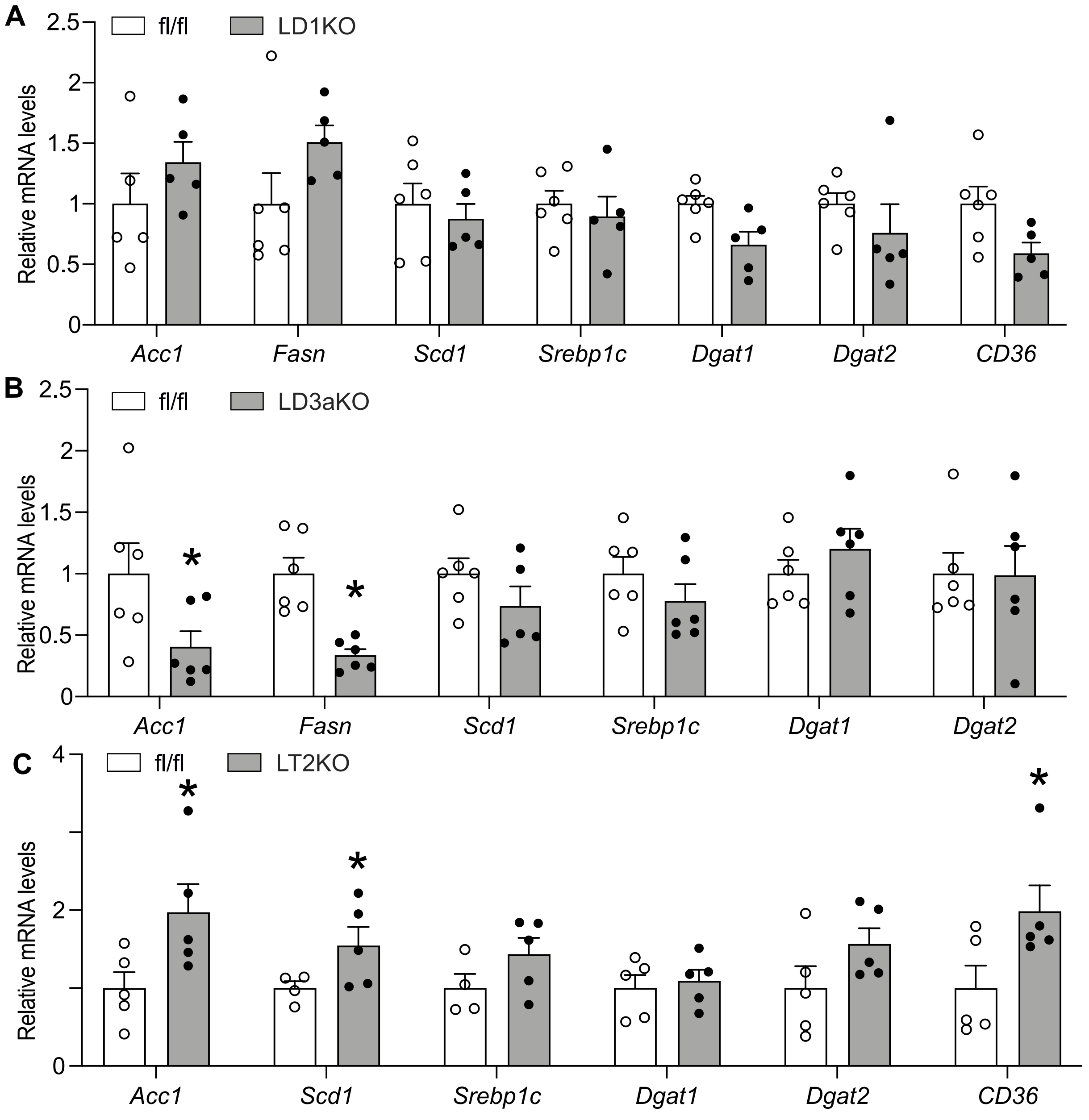

Supplemental figure 13

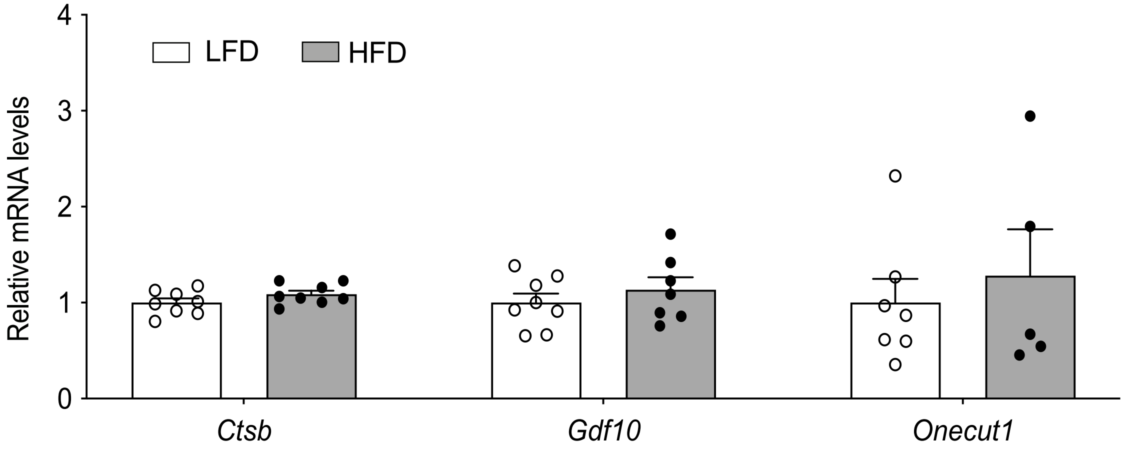

Supplemental figure 14

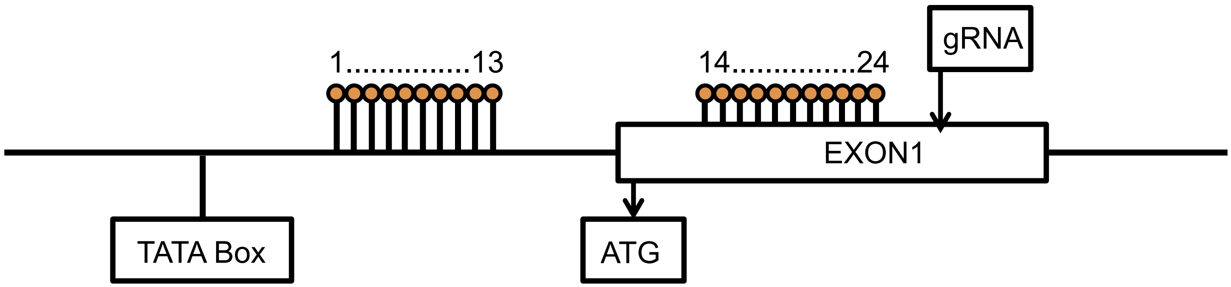

Supplemental figure 15

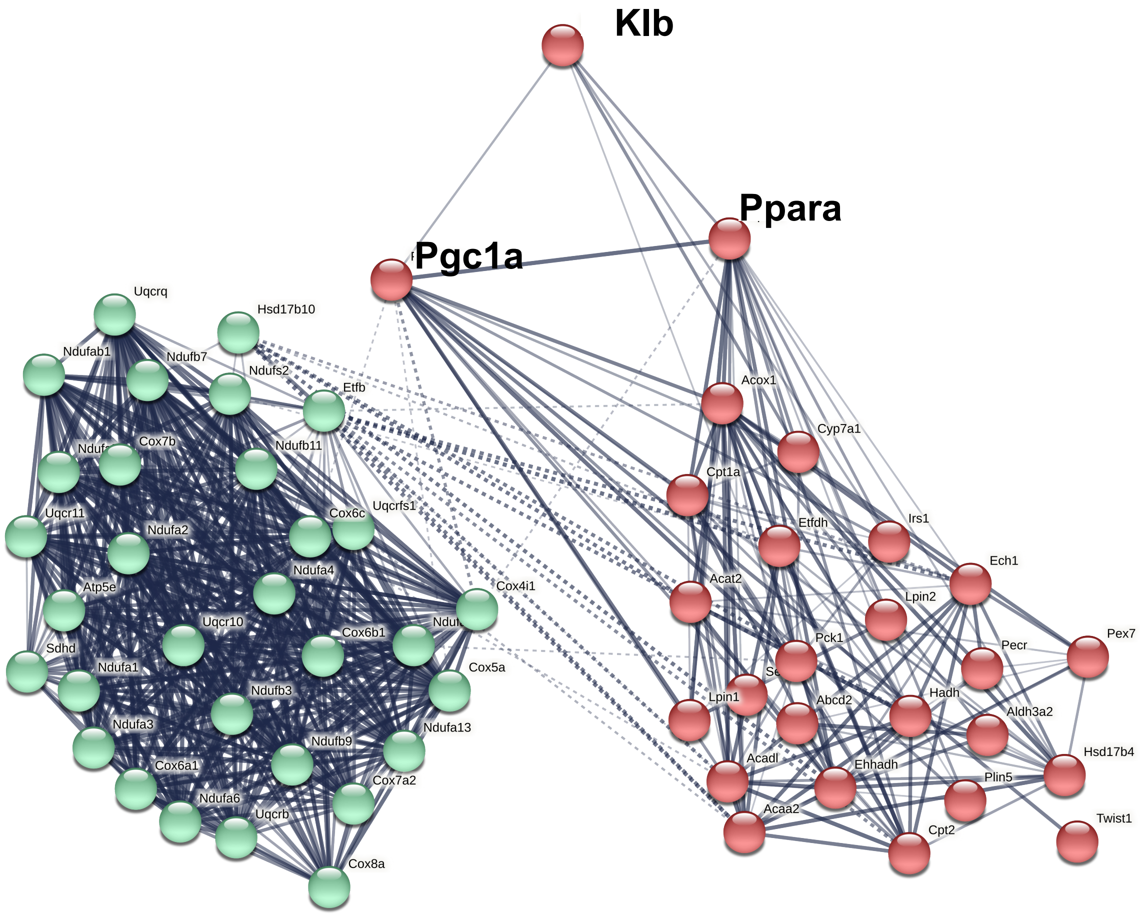

Supplemental figure 16

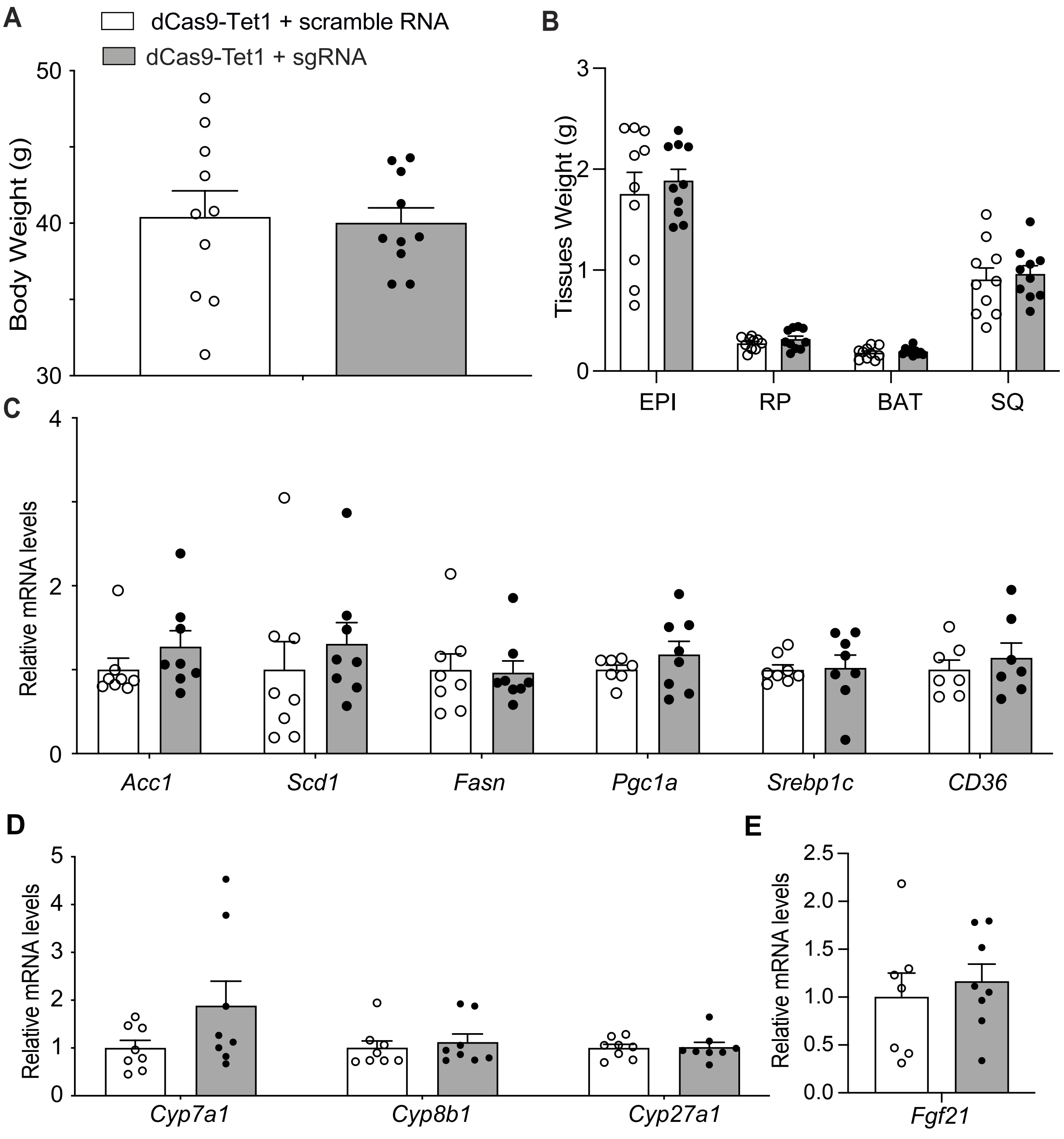

Supplemental figure 17

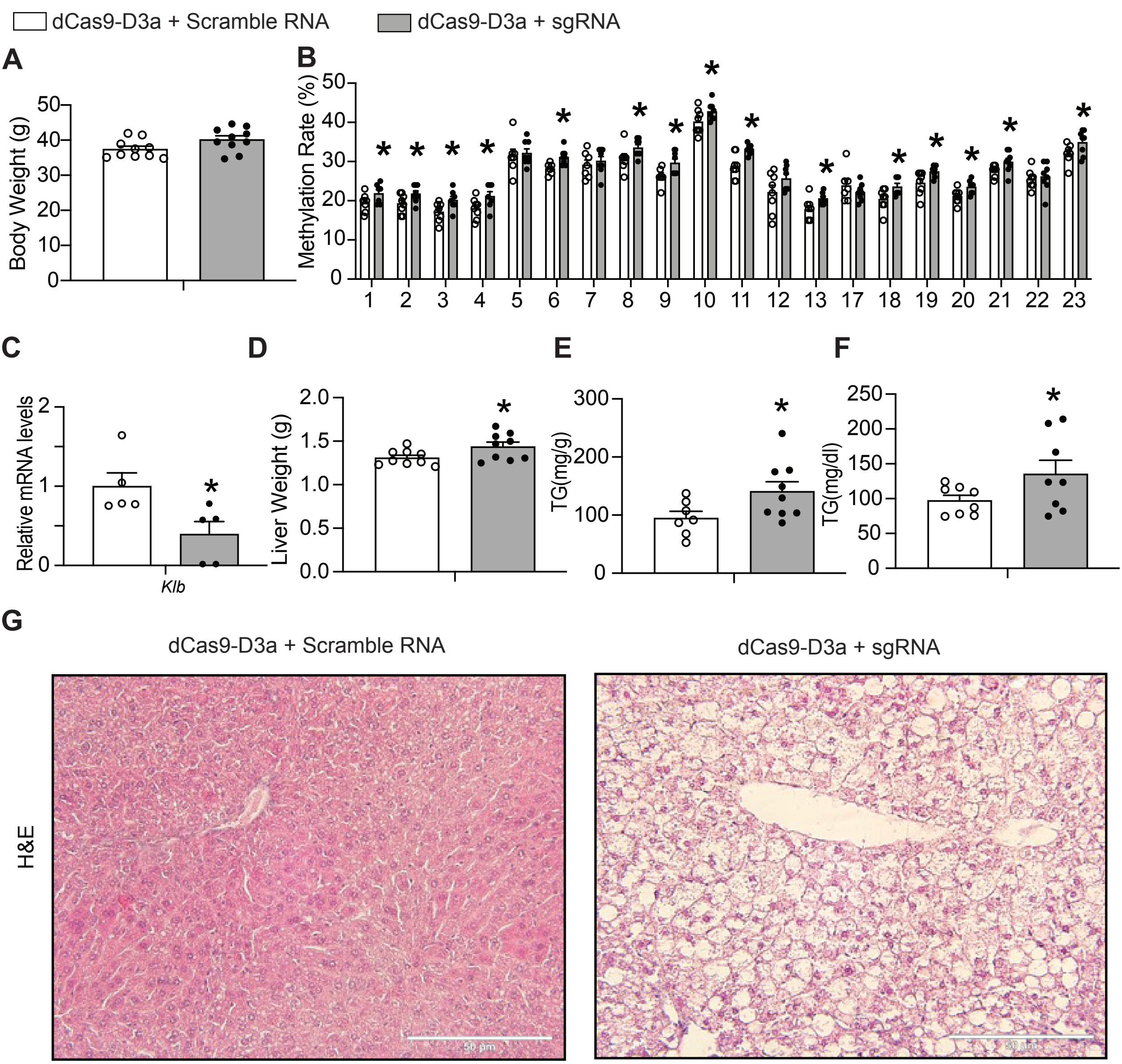

Supplementary Table 1: The list of genes in the Differentially Methylated Regions (DMR)

Chromoso	Starting po	End positio	p-value	Methylation ratio of HF/LF	
chr11	11925660	11925740	2.38E-05	2.00	NM_001177629
chr11	11925660	11925740	2.38E-05	2.00	NM_010345
chr14	63860465	63860648	3.57E-05	3.03	NM_008092
chr19	7496676	7496754	1.45E-04	2.48	NM_175381
chr16	78335085	78335179	4.38E-08	3.59	NM_001025192
chr16	78335085	78335179	4.38E-08	3.59	NM_009988
chr15	89354427	89354572	3.50E-30	2.09	NM_021423
chr4	1.4E+08	1.4E+08	3.18E-103	2.03	NM_173427
chr11	70279513	70279574	2.37E-13	2.31	NM_001029929
chr17	85187901	85187976	1.53E-03	2.68	NM_028233
chr1	1.29E+08	1.29E+08	3.40E-10	2.06	NM_145128
chr7	31505043	31505114	9.07E-40	2.06	NM_018731
chr7	31895897	31895984	2.17E-17	2.00	NM_008281
chr7	31895897	31895984	2.17E-17	2.00	NM_001110252
chr11	31865777	31865919	1.71E-09	2.48	NM_026262
chr7	52894146	52894222	1.32E-16	2.15	NM_028544
chr6	1.25E+08	1.25E+08	1.48E-08	3.06	NM_009415
chr5	36673487	36673648	2.58E-12	2.25	NM_030889
chr11	50527466	50527585	2.42E-04	2.30	NM_175643
chr11	96194056	96194369	3.18E-125	2.60	NM_001079869
chr11	74979953	74980020	1.13E-23	2.04	NM_001098203
chr11	74979953	74980020	1.13E-23	2.04	NM_010430
chr17	34974571	34975018	5.32E-30	2.08	NM_001163770
chr17	34974571	34975018	5.32E-30	2.08	NM_033613
chr3	95697902	95698034	2.72E-77	4.17	NM_146104
chr3	95697902	95698034	2.72E-77	4.17	NM_146134
chr17	56263664	56263715	3.80E-50	2.04	NM_013662
chr17	56263664	56263715	3.80E-50	2.04	NM_001130456
chr3	95231781	95232120	1.24E-07	3.27	NR_033637
chr8	86654925	86654988	5.51E-34	2.04	NM_001013384
chr14	66378499	66378543	3.00E-13	2.09	NM_028903
chr2	29105693	29105871	1.29E-16	2.04	NR_029460
chr3	94976617	94976678	1.05E-05	2.47	NM_011351
chr5	65739507	65739914	6.26E-05	2.24	NM_031180
chr7	1.13E+08	1.13E+08	6.67E-64	2.04	NM_001162943
chr8	73340726	73340858	2.74E-69	4.70	NM_008353
chr17	89191189	89191316	1.27E-14	2.25	NM_013582
chr6	1.08E+08	1.08E+08	1.95E-03	2.65	NM_145937
chr6	1.26E+08	1.26E+08	2.36E-11	2.05	NM_145983
chr18	37926434	37926484	7.33E-04	3.67	NM_001174154
chr18	37926434	37926484	7.33E-04	3.67	NM_033584
chr18	37926434	37926484	7.33E-04	3.67	NM_033585
chr18	37926434	37926484	7.33E-04	3.67	NM_033586
chr18	37926434	37926484	7.33E-04	3.67	NM_033574

chr18	37926434	37926484	7.33E-04	3.67	NM_033587
chr18	37926434	37926484	7.33E-04	3.67	NM_033575
chr18	37926434	37926484	7.33E-04	3.67	NM_033588
chr18	37926434	37926484	7.33E-04	3.67	NM_033589
chr18	37926434	37926484	7.33E-04	3.67	NM_033590
chr18	37926434	37926484	7.33E-04	3.67	NM_033576
chr18	37926434	37926484	7.33E-04	3.67	NM_033591
chr18	37926434	37926484	7.33E-04	3.67	NM_033577
chr18	37926434	37926484	7.33E-04	3.67	NM_033592
chr18	37926434	37926484	7.33E-04	3.67	NM_033578
chr18	37926434	37926484	7.33E-04	3.67	NM_033593
chr18	37926434	37926484	7.33E-04	3.67	NM_033579
chr18	37926434	37926484	7.33E-04	3.67	NM_033594
chr18	37926434	37926484	7.33E-04	3.67	NM_033580
chr18	37926434	37926484	7.33E-04	3.67	NM_033595
chr2	35517445	35517603	1.71E-17	2.10	NM_001114125
chr2	35517445	35517603	1.71E-17	2.10	NM_001114124
chr10	79616408	79616526	6.22E-12	3.58	NM_021565
chr7	31374877	31375293	5.17E-28	2.05	NM_021397
chr11	1.03E+08	1.03E+08	5.04E-09	2.80	NM_001077698
chr11	1.03E+08	1.03E+08	5.04E-09	2.80	NM_019679
chr7	19608258	19608402	1.94E-55	2.21	NM_008260
chr11	49074557	49074767	1.10E-07	2.11	NM_010794
chr11	49074557	49074767	1.10E-07	2.11	NM_001110148
chr11	49074557	49074767	1.10E-07	2.11	NM_001110149
chr11	49074557	49074767	1.10E-07	2.11	NM_001110150
chr17	84586822	84586953	4.83E-06	1.95	NM_001001806
chr8	86545092	86545427	8.73E-12	2.17	NM_028877
chr1	75483776	75483864	1.67E-05	2.00	NM_178884
chr11	48982896	48983043	4.89E-47	2.00	NM_172793
chr11	87952814	87952874	1.80E-10	2.00	NM_198013
chr11	1.02E+08	1.02E+08	1.89E-79	2.11	NM_199200
chr11	1.2E+08	1.2E+08	1.09E-93	2.53	NM_009609
chr17	32533377	32533448	1.03E-09	2.24	NM_178785
chr4	1.15E+08	1.15E+08	2.57E-08	2.00	NM_008593
chr3	1.22E+08	1.22E+08	1.60E-07	3.08	NM_013867
chr17	47638444	47638565	2.72E-05	2.50	NM_022015
chr4	1.48E+08	1.48E+08	1.84E-22	1.94	NM_001159344
chr7	90017657	90017771	2.05E-21	2.19	NM_175366
chr4	53727191	53727450	2.11E-115	6.80	NM_139309
chr4	1.23E+08	1.23E+08	3.47E-10	2.00	NM_007558
chr4	1.23E+08	1.23E+08	3.47E-10	2.00	NM_022033
chr1	74074119	74074261	2.92E-23	3.67	NM_027884
chr10	1.27E+08	1.27E+08	5.98E-124	2.54	NM_001033263
chr15	1.01E+08	1.01E+08	3.76E-21	2.06	NM_033073
chr8	86568701	86568749	4.49E-29	2.06	NM_173184
chr13	1.14E+08	1.14E+08	1.76E-16	4.57	NM_130796

chr4	1.35E+08	1.35E+08	1.96E-20	2.13	NM_019732
chr14	22804239	22804287	6.80E-12	2.21	NM_145459
chr14	70479081	70479323	3.41E-11	2.07	NM_018781
chr16	95930275	95930429	2.08E-03	2.21	NM_011809
chr3	1.05E+08	1.05E+08	8.17E-12	2.58	NM_007484
chr5	1.35E+08	1.35E+08	2.91E-16	2.07	NM_010876
chr8	1.08E+08	1.08E+08	4.55E-239	3.10	NM_001033320
chr9	20446485	20446642	8.39E-05	2.07	NM_013911
chr9	20446485	20446642	8.39E-05	2.07	NM_001002846
chr11	82853464	82853579	1.69E-05	2.50	NM_181542
chr11	96206974	96207326	1.27E-33	2.31	NM_010458
chr16	92695942	92696280	1.84E-06	2.31	NM_009821
chr16	92695942	92696280	1.84E-06	2.31	NM_001111023
chr16	92695942	92696280	1.84E-06	2.31	NM_001111022
chr16	92695942	92696280	1.84E-06	2.31	NM_001111021
chr11	1.17E+08	1.17E+08	4.77E-23	2.07	NM_001113486
chr6	52153852	52154262	0.00E+00	3.22	NR_015611
chr6	52153852	52154262	0.00E+00	3.22	NM_010453
chr7	25162145	25162533	1.18E-69	2.64	NM_008433
chr7	25162145	25162533	1.18E-69	2.64	NM_001163510
chr8	73122388	73122469	2.52E-36	1.93	NM_133772
chr1	1.36E+08	1.36E+08	4.41E-26	2.15	NM_001144855
chr11	58990739	58990822	7.60E-16	2.15	NM_175452
chr11	58990739	58990822	7.60E-16	2.15	NM_080454
chr11	88007305	88007379	6.39E-12	2.15	NM_001172099
chr16	10796757	10796811	6.34E-10	2.15	NM_013637
chr19	61302174	61302244	1.71E-09	2.15	NM_009970
chr4	1.17E+08	1.17E+08	2.51E-67	2.80	NM_025739
chr1	91825416	91825563	2.70E-24	2.25	NM_010262
chr2	1.32E+08	1.32E+08	1.36E-05	2.08	NM_023043
chr2	1.32E+08	1.32E+08	1.36E-05	2.08	NM_001126338
chr6	52140440	52140591	4.18E-24	2.08	NM_008265
chr6	1.19E+08	1.19E+08	5.22E-03	2.70	NM_001033382
chr6	1.19E+08	1.19E+08	5.22E-03	2.70	NM_001172207
chr6	1.19E+08	1.19E+08	5.22E-03	2.70	NM_172492
chr1	1.08E+08	1.08E+08	2.33E-09	2.36	NM_009399
chr16	35660150	35660591	3.89E-139	3.71	NM_013661
chr2	1.27E+08	1.27E+08	2.04E-04	6.50	NM_178047
chr2	1.27E+08	1.27E+08	2.04E-04	6.50	NM_138750
chr2	1.47E+08	1.47E+08	5.99E-07	2.17	NM_001077632
chr2	1.47E+08	1.47E+08	5.99E-07	2.17	NM_010919
chr11	84333789	84333847	1.72E-05	2.08	NM_008498
chr2	1.32E+08	1.32E+08	3.12E-11	5.00	NM_018824
chr6	1.26E+08	1.26E+08	2.19E-04	3.13	NM_001164035
chr6	1.26E+08	1.26E+08	2.19E-04	3.13	NM_001164034
chr6	1.26E+08	1.26E+08	2.19E-04	3.13	NM_008742

chr13	64264085	64264346	6.29E-08	2.18	NM_019986
chr19	8845550	8845656	3.38E-04	2.18	NM_025791
chr9	58159834	58159995	3.77E-23	2.18	NM_010729
chr2	37631803	37631897	3.14E-07	2.56	NM_001163566
chr4	1.29E+08	1.29E+08	8.49E-11	2.09	NM_001085491
chr8	19892594	19892667	5.14E-26	4.60	NR_030708
chr11	70333941	70334236	5.64E-07	2.75	NM_146020
chr2	1.19E+08	1.19E+08	7.18E-06	2.20	NM_177568
chr5	1.08E+08	1.08E+08	1.79E-63	2.44	NM_010278
chr10	1.27E+08	1.27E+08	1.15E-40	2.10	NM_146011
chr17	56609309	56609428	1.25E-10	2.10	NM_011218
chr17	57226968	57227015	2.17E-13	2.10	NM_009451
chr5	77444818	77445136	2.74E-62	4.20	NM_027970
chr7	19542973	19543130	3.02E-26	2.10	NM_001029877
chr7	52109226	52109406	7.85E-03	2.33	NM_026270
chr7	69609128	69609203	1.11E-05	1.91	NM_013788
chr8	3392960	3393130	2.28E-102	3.00	NM_133962
chr2	1.3E+08	1.3E+08	6.35E-07	2.00	NM_001110513
chr5	77286456	77286749	5.60E-07	2.50	NM_001163793
chr10	85061534	85061664	8.43E-06	2.38	NM_028709
chr10	85061534	85061664	8.43E-06	2.38	NM_001017525
chr11	3271274	3271527	2.74E-06	2.38	NM_173053
chr11	3271274	3271527	2.74E-06	2.38	NM_010718
chr16	45844301	45844531	1.05E-57	3.60	NM_153412
chr4	1.45E+08	1.45E+08	2.27E-41	2.00	NR_002888
chr7	1.51E+08	1.51E+08	2.55E-06	3.00	NM_024289
chr9	74716490	74716628	5.04E-04	2.57	NM_008262
chr12	87039318	87039384	3.51E-25	2.13	NM_016767
chr12	88223381	88223636	6.40E-10	4.25	NM_145836
chr12	1.14E+08	1.14E+08	3.21E-16	2.43	NM_153776
chr2	1.64E+08	1.64E+08	6.57E-10	2.13	NM_008435
chr4	1.33E+08	1.33E+08	6.80E-31	2.83	NM_027152
chr7	53653479	53653555	2.65E-08	2.13	NM_008421
chr7	53653479	53653555	2.65E-08	2.13	NM_001112739
chr7	1.28E+08	1.28E+08	2.13E-26	1.89	NR_024051
chr8	48373343	48373574	1.47E-40	3.40	NM_001114311
chr8	48373343	48373574	1.47E-40	3.40	NM_175162
chr1	1.83E+08	1.83E+08	1.99E-08	4.00	NM_010094
chr11	1.2E+08	1.2E+08	4.37E-06	2.00	NR_030682
chr17	23879637	23879742	6.62E-08	2.29	NM_028416
chr5	1.38E+08	1.38E+08	1.71E-03	5.33	NM_007942
chr12	58643626	58643685	1.60E-13	2.14	NM_008259
chr4	59047994	59048442	0.00E+00	7.50	NM_025277
chr4	1.3E+08	1.3E+08	3.16E-12	2.14	NM_173071
chr6	52125626	52125719	1.03E-18	2.14	NM_010452
chr1	1.68E+08	1.68E+08	7.57E-11	2.00	NM_001164528

chr10	94877028	94877098	1.34E-06	2.00	NM_001168657
chr10	94877028	94877098	1.34E-06	2.00	NM_001168656
chr10	94877028	94877098	1.34E-06	2.00	NM_001168655
chr10	94877028	94877098	1.34E-06	2.00	NM_007706
chr13	94444280	94444470	4.15E-30	2.33	NM_028772
chr15	1.03E+08	1.03E+08	5.46E-05	3.50	NM_010466
chr7	1.12E+08	1.12E+08	2.70E-17	2.00	NM_199146
chr7	1.12E+08	1.12E+08	2.70E-17	2.00	NM_001167828
chr11	1.02E+08	1.02E+08	1.86E-03	2.17	NM_020510
chr3	96177757	96177817	1.54E-04	4.33	NR_024200
chrX	71069170	71069272	2.13E-12	2.17	NM_001029868
chr14	34737255	34737331	7.51E-05	4.00	NM_145741
chr2	1.68E+08	1.68E+08	1.03E-10	2.00	NM_001160330
chr2	1.8E+08	1.8E+08	1.72E-12	2.40	NM_178254
chr5	24031814	24031913	3.75E-07	2.40	NM_015739
chr7	25422147	25422264	3.41E-06	2.00	NM_133743
chr1	1.87E+08	1.87E+08	1.30E-32	2.75	NM_001081361
chr11	45793776	45793865	1.64E-04	2.20	NM_173384
chr7	29076020	29076082	1.62E-05	2.20	NM_175033
chr7	29079529	29079622	3.47E-06	1.83	NM_007866
chr7	1.52E+08	1.52E+08	4.57E-15	5.50	NM_007803
chr12	77810253	77810497	1.03E-03	2.00	NM_013675
chr19	16511129	16511246	8.05E-07	2.00	NM_008137
chr3	99057940	99058116	4.11E-06	3.33	NM_009323
chr7	29252194	29252673	2.05E-26	2.00	NM_001141921
chr8	72635998	72636043	1.05E-03	2.00	NM_007789
chr17	49099601	49099782	5.80E-13	4.50	NM_027452
chr19	18745505	18745624	5.73E-05	2.25	NM_026120
chr2	1.48E+08	1.48E+08	3.82E-06	2.25	NM_009219
chr5	1.5E+08	1.5E+08	4.05E-06	4.50	NM_027519
chr8	1.13E+08	1.13E+08	2.17E-04	2.25	NM_007586
chr12	12946328	12946555	8.37E-05	2.67	NM_008709
chr13	12432623	12432746	4.35E-04	2.67	NM_033268
chr15	75839379	75839763	1.27E-17	2.67	NM_001168253
chr15	75839379	75839763	1.27E-17	2.67	NM_134087
chr18	77095099	77095192	8.46E-06	2.00	NM_001109743
chr2	1.52E+08	1.52E+08	2.78E-04	2.00	NM_175126
chr3	1.08E+08	1.08E+08	3.76E-07	2.00	NM_008596
chr3	1.21E+08	1.21E+08	1.44E-06	2.00	NM_145394
chr4	1.54E+08	1.54E+08	8.29E-05	2.67	NM_027504
chr4	1.54E+08	1.54E+08	8.29E-05	2.67	NM_001177995
chr8	95379076	95379314	7.95E-07	2.67	NM_173014
chr12	1.2E+08	1.2E+08	3.42E-08	2.33	NM_177082
chr3	87559935	87560081	1.73E-09	7.00	NM_152799
chr3	87559935	87560081	1.73E-09	7.00	NM_001032413
chr3	87559935	87560081	1.73E-09	7.00	NM_028460

chr3	87559935	87560081	1.73E-09	7.00	NM_001032414
chr6	90686182	90686360	4.06E-07	1.75	NM_001134384
chr7	88359125	88359182	2.22E-05	2.33	NM_008803
chr1	38683791	38684002	2.49E-09	2.00	NM_010678
chr14	61792111	61792262	2.55E-10	6.00	NM_172809
chr15	89209102	89209154	1.10E-05	2.00	NM_001013022
chr17	80772686	80772740	4.21E-04	2.00	NM_001145452
chr17	80772686	80772740	4.21E-04	2.00	NR_028385
chr2	1.72E+08	1.72E+08	1.17E-05	2.00	NM_175631
chr4	44676977	44677074	6.43E-05	2.00	NM_008782
chr8	1.28E+08	1.28E+08	1.11E-03	2.00	NM_028908
chr11	94254115	94254245	1.70E-05	2.50	NM_029600
chr13	54839576	54839643	1.15E-04	2.50	NM_012014
chr16	21205328	21205389	5.89E-06	1.67	NM_010143
chr18	80519298	80519343	2.36E-03	2.50	NM_001190373
chr2	29050497	29050732	8.17E-05	2.50	NM_133500
chr2	29050497	29050732	8.17E-05	2.50	NM_133501
chr2	70400259	70400372	1.18E-05	2.50	NM_008077
chr2	74495948	74496001	4.88E-04	2.50	NM_007967
chr5	15440972	15441133	1.22E-03	2.50	NM_001110846
chr5	15440972	15441133	1.22E-03	2.50	NM_009784
chr5	15440972	15441133	1.22E-03	2.50	NM_001110844
chr5	15440972	15441133	1.22E-03	2.50	NM_001110843
chr5	15440972	15441133	1.22E-03	2.50	NM_001110845
chr5	30559453	30559510	1.00E-07	5.00	NM_027652
chr5	1.47E+08	1.47E+08	1.87E-12	1.67	NM_145155
chr7	86040779	86040973	2.52E-21	5.00	NM_026531
chr7	86040779	86040973	2.52E-21	5.00	NM_001162939
chr7	91031478	91031643	2.39E-06	2.50	NM_030705
chr7	1.27E+08	1.27E+08	7.73E-07	2.50	NM_026458
chr7	1.29E+08	1.29E+08	2.57E-04	2.50	NM_001081327
chr7	1.4E+08	1.4E+08	2.87E-08	2.50	NM_016978
chr8	32200272	32200399	1.59E-05	2.50	NM_025869
chr10	80730891	80731031	5.63E-06	4.00	NM_018758
chr10	81510966	81511145	1.65E-18	4.00	NM_001024626
chr11	1.03E+08	1.03E+08	2.17E-05	2.00	NM_183288
chr12	77518497	77518679	3.94E-06	4.00	NM_001163103
chr12	81859776	81859880	3.48E-06	2.00	NM_001008423
chr14	55195261	55195408	2.42E-06	2.00	NM_010590
chr16	4756953	4757095	2.35E-06	4.00	NM_001136066
chr16	4756953	4757095	2.35E-06	4.00	NM_010443
chr17	34164963	34165038	1.98E-03	2.00	NM_013543
chr2	1.7E+08	1.7E+08	3.33E-03	2.00	NM_009996
chr2	1.7E+08	1.7E+08	3.33E-03	2.00	NM_001013369
chr3	9250801	9250866	3.44E-07	2.00	NM_177660
chr4	1.11E+08	1.11E+08	2.36E-03	4.00	NM_026470

chr7	15208098	15208161	1.30E-07	2.00	NM_001039146
chr7	64765116	64765161	3.79E-04	2.00	NM_176942
chr7	1.22E+08	1.22E+08	8.40E-10	4.00	NM_177382
chr8	24522552	24522661	8.48E-07	4.00	NM_013834
chr8	1.24E+08	1.24E+08	6.65E-05	2.00	NM_080855
chr9	98856249	98856333	1.10E-02	2.00	NM_012020
chr1	64737835	64737991	3.58E-06	3.00	NM_001097644
chr11	54891476	54891712	1.37E-10	3.00	NM_177471
chr12	56700657	56700759	3.12E-05	1.50	NM_020287
chr12	74647005	74647096	1.78E-05	3.00	NM_178715
chr13	55927450	55927558	4.90E-06	1.50	NM_011097
chr15	93105327	93105386	4.07E-07	3.00	NM_001033275
chr17	13185190	13185306	2.57E-06	3.00	NM_175394
chr5	1.32E+08	1.32E+08	3.83E-05	3.00	NM_177047
chr7	72300576	72300738	5.81E-05	3.00	NM_183087
chr7	1.29E+08	1.29E+08	7.19E-05	1.50	NM_008855
chr8	87083414	87083468	9.94E-05	3.00	NM_007578
chr8	1.23E+08	1.23E+08	1.60E-03	3.00	NM_001163762
chr8	1.23E+08	1.23E+08	1.60E-03	3.00	NM_172286
chr8	1.23E+08	1.23E+08	1.60E-03	3.00	NM_001163761
chr10	1.23E+08	1.23E+08	8.49E-03	2.00	NM_182807
chr12	70683709	70683938	6.30E-22	2.00	NM_001033236
chr2	33487029	33487154	7.30E-04	2.00	NM_010725

Chromoso	Starting po	End positio	Methylation ratio of HF/LF	
chr14	70591590	70591652	0.50	NM_011366
chr2	1.3E+08	1.3E+08	0.48	NM_197945
chr15	25703712	25704052	0.46	NM_019472
chr7	31278995	31279115	0.47	NM_019546
chr10	55834959	55835061	0.30	NM_001033385
chr10	55834959	55835061	0.30	NM_001163833
chr19	5726140	5726233	0.45	NM_001114597
chr19	5726140	5726233	0.45	NM_001114595
chr19	5726140	5726233	0.45	NM_001114596
chr19	5726140	5726233	0.45	NM_053252
chr8	72612182	72612238	0.36	NM_177900
chr11	1.17E+08	1.17E+08	0.38	NM_001113486
chr11	1.17E+08	1.17E+08	0.38	NM_001113487
chr7	1.51E+08	1.51E+08	0.43	NM_001113373
chr17	36257810	36257893	0.44	NM_010395
chr5	1.3E+08	1.3E+08	0.09	NM_027121
chr5	1.3E+08	1.3E+08	0.09	NM_001001327
chr6	1.48E+08	1.48E+08	0.37	NM_198967
chr6	1.48E+08	1.48E+08	0.37	NR_003634
chr4	1.29E+08	1.29E+08	0.19	NM_153541
chr6	1.28E+08	1.28E+08	0.35	NM_175414
chr11	76384410	76384564	0.35	NM_198895
chr11	76384410	76384564	0.35	NM_198894
chr5	1.15E+08	1.15E+08	0.40	NM_133904
chr4	1.18E+08	1.18E+08	0.37	NM_009711
chr6	1.27E+08	1.27E+08	0.06	NM_022657
chr15	99287539	99287669	0.33	NM_001001884
chr11	74980566	74980660	0.21	NM_001098203
chr11	74980566	74980660	0.21	NM_010430
chr17	24670379	24670472	0.50	NM_177375
chr5	1.45E+08	1.45E+08	0.43	NM_016789
chr7	28114534	28114622	0.21	NM_001113549
chr7	28114534	28114622	0.21	NM_175641
chr14	66572507	66572611	0.42	NM_172604
chr5	1.39E+08	1.39E+08	0.50	NM_008808
chr8	47377614	47377815	0.33	NM_173789
chrX	11710606	11710769	0.33	NM_001168321
chr13	3477505	3477642	0.20	NR_015522
chr13	47109694	47109846	0.30	NM_175340
chr5	66651474	66651553	0.50	NM_027602
chr11	96181376	96181454	0.44	NM_010459
chr4	1.31E+08	1.31E+08	0.33	NM_001083119
chr4	1.31E+08	1.31E+08	0.33	NM_011214
chr8	3516888	3517094	0.11	NM_001122818
chr8	3516888	3517094	0.11	NM_015801

chr19	41817823	41817917	0.50	NM_015748
chr7	50862583	50862794	0.38	NM_028913
chr8	87083293	87083353	0.38	NM_007578
chr9	20988606	20988708	0.50	NM_183408
chr2	1.19E+08	1.19E+08	0.43	NM_016907
chr4	1.3E+08	1.3E+08	0.43	NM_198959
chr6	85434282	85434331	0.43	NM_001001160
chr2	92241245	92241311	0.50	NM_011162
chr9	20446637	20446804	0.33	NM_013911
chr9	20446637	20446804	0.33	NM_001002846
chr15	75396942	75397097	0.40	NM_001135689
chr15	75396942	75397097	0.40	NM_001135688
chr15	75396942	75397097	0.40	NM_011837
chr1	94728913	94729061	0.25	NM_016696
chr11	1.17E+08	1.17E+08	0.25	NM_172948
chr14	1.23E+08	1.23E+08	0.50	NM_009574
chr4	1.37E+08	1.37E+08	0.50	NM_001081155
chr6	24905804	24905920	0.50	NM_177013
chr8	12757548	12757601	0.25	NM_015804
chr1	34293471	34293609	0.33	NM_133833
chr1	34293471	34293609	0.33	NM_134448
chr11	45794048	45794154	0.33	NM_173384
chr13	36061086	36061391	0.33	NM_029628
chr13	48758461	48758553	0.67	NM_007526
chr15	74346918	74346977	0.33	NM_174991
chr19	36992893	36993183	0.33	NM_028263
chr2	93850797	93850956	0.33	NM_026944
chr2	1.51E+08	1.51E+08	0.33	NM_008019
chr4	1.32E+08	1.32E+08	0.33	NM_001128607
chr4	1.32E+08	1.32E+08	0.33	NM_001128606
chr4	1.32E+08	1.32E+08	0.33	NM_183428
chr5	1.32E+08	1.32E+08	0.33	NM_177047
chr12	1.1E+08	1.1E+08	0.50	NM_001163175
chr2	3035940	3036050	0.50	NM_001081161
chr2	35437571	35437624	0.50	NM_001114125
chr5	8422808	8422945	0.50	NM_178766
chr7	31935880	31936098	0.50	NM_027898
